# Epigenetic regulation of intestinal peptide transporter PEPT1 as a potential strategy for colorectal cancer sensitization

**DOI:** 10.1038/s41419-021-03814-5

**Published:** 2021-05-24

**Authors:** Yanhong Wang, Jiaqi Wang, Lingrong Yang, Liqing Qiu, Yuhui Hua, Shixiu Wu, Su Zeng, Lushan Yu, Xiaoli Zheng

**Affiliations:** 1 Institute of Drug Metabolism and Pharmaceutical Analysis, Zhejiang Province Key Laboratory of Anti-Cancer Drug Research, College of Pharmaceutical Sciences, Zhejiang University, 310058 Hangzhou, China; 2 Department of Pharmacy, Hangzhou Cancer Hospital, 310002 Hangzhou, China; 3 National Cancer Center/National Clinical Research Center for Cancer/Cancer Hospital & Shenzhen Hospital, Chinese Academy of Medical Sciences and Peking Union Medical College, 518116 Shenzhen, China

**Keywords:** Molecular biology, Gene silencing

## Abstract

Human intestinal peptide transporter PEPT1 is commonly repressed in human colorectal cancer (CRC), yet its relationship with sensitivity to the common CRC treatment ubenimex has not previously been elucidated. In this study, we confirmed PEPT1 suppression in CRC using real-time quantitative polymerase chain reaction and western blotting and then investigated the underlying epigenetic pathways involved using bisulfite sequencing, chromatin immunoprecipitation, siRNA knockdown, and reporter gene assays. We found that *PEPT1* transcriptional repression was due to both DNMT1-mediated DNA methylation of the proximal promoter region and HDAC1-mediated histone deacetylation, which blocked P300-mediated H3K18/27Ac at the *PEPT1* distal promoter. Finally, the effects of the epigenetic activation of PEPT1 on the CRC response to ubenimex were evaluated using sequential combination therapy of decitabine and ubenimex both in vitro and in xenografts. In conclusion, epigenetic silencing of PEPT1 due to increased DNMT1 and HDAC1 expression plays a vital role in the poor response of CRC to ubenimex.

## Introduction

Colorectal cancer (CRC) is the third most commonly diagnosed cancer and the second most deadly cancer worldwide, with an estimated over 1.9 million new cases and 935,000 deaths recorded in 2020^1^. CRC incidence and mortality have declined with recent screening programs. However, the diagnosis rates of advanced CRC with low overall survival (OS) remain high^1^. Therefore, it is essential to identify novel biomarkers for the treatment, diagnosis, and prognosis of CRC. Chemotherapy is the treatment of choice to increase the OS of patients with advanced or metastatic CRC^2^. The resistance of malignant tumor cells to chemotherapy is a crucial reason for poor survival among CRC patients. One explanation for this multidrug resistance (MDR) to cancer chemotherapy is the increased efflux and reduced influx mediated by drug transporters^3^. For example, our laboratory’s previous studies have demonstrated that the repression of uptake transporters resulted in decreased drug concentrations in tumors^4,5^.

The peptide transporter PEPT1 (encoded by *SLC15A1*) is a prototypical member of the SLC15 family^6^. PEPT1 is predominantly responsible for the absorption of di/tripeptides and is mainly located on brush border membranes of small intestinal epithelia. In addition to that in the intestine, PEPT1 has been detected in tissues such as the nasal epithelium, kidney, biliary duct, and macrophages^7,8^. Besides, PEPT1 is overexpressed in the colon of inflammatory bowel disease patients and prostate cancer cells, which provides novel insight into the pathogenesis and tumor-specific drug delivery of this diseases^9,10^. However, there are inconsistent reports on the expression levels of PEPT1 mRNA and protein in the colon^11^. While one study reported low PEPT1 expression in the colon^12^, it could not be detected in other studies^9,13^. Another recent study showed that PEPT1 is highly expressed in the distal colon^14^. These inconsistent results may be explained at least partially by the different research groups measuring expression in different areas of the colon. PEPT1 has considerable substrate specificity for oligopeptides produced after the digestion of dietary or body proteins and structurally related drugs, such as the anticancer agent ubenimex (UBEN) and peptidomimetic prodrugs^15^. Ubenimex (UBEN), more commonly known as Bestatin, is a drug with immune-modulatory and anti-tumor activities^16^. At present, it has been widely used to treat acute myelocytic leukemia^17^ and it was once reportedly, preferably delivered into tumor cells overexpressing PEPT1^18^. Therefore, PEPT1 is increasingly becoming a potential target for modulating the efficacy of various chemotherapeutic agents.

Until now, the transcriptional regulation of PEPT1 has not been widely investigated. Only a few transcription factors (sp1, cdx2, nrf2) have been shown to regulate the transcriptional activity of the PEPT1 gene promoter^19–21^. Epigenetic regulation of gene transcription, which involves dynamic modifications such as DNA methylation, histone acetylation, and noncoding RNAs, has been associated with several physiological and pathological processes. Although DNA methylation typically involved in gene repression is performed by DNA methyltransferase (DNMT1, DNMT3a, and DNMT3b), DNMT1 is primarily responsible for maintenance methylation by converting hemimethylated duplexes into symmetrically methylated CpG dinucleotides during DNA replication^22,23^. Alterations in DNA methylation caused by abnormalities in DNMT1 have been shown to drive tumorigenesis in several studies^24,25^. Histone acetylation primarily occurs at numerous lysine residues at the N terminus of histones, including H3K9Ac, H3K18Ac, and H3K27Ac, which are enriched around the transcription start site (TSS) and are generally associated with gene activation^26,27^. Acetyl groups are added at these specific histone sites by histone acetyltransferases (HATs) and removed by histone deacetylases (HDACs)^28^. The four main groups of HATs are the GCN5, MYST, CBP/P300, and SRC/p160 nuclear receptor coactivator families, and the HDAC family comprises eighteen different isoforms (HDAC1-11 and SIRT1-7)^28^. Similar to DNA methylation, histone acetylation at various gene transcriptional regulatory elements has been correlated with tumor development^29^. Epigenetic instability in CRC occurs early and manifests more frequently than genetic modifications^30^. For example, DNMT1-mediated methylation of *Cdknla* promoted cell proliferation in a carcinogen-administered CRC mouse model^31^. In addition, P300 was shown to promote PHF5A acetylation at K29 during cellular stress, which consequently contributed to colon carcinogenesis^32^ in the CRC xenograft model. Here, we determined the expression level of PEPT1 in CRC compared to normal tissues and analyzed the epigenetic mechanisms deregulating the expression of PEPT1 in CRC. We then designed a combination treatment of decitabine (DAC) and UBEN to sensitize CRC cells to UBEN. These findings highlight the potential clinical usefulness of various epigenetic modifications as biomarkers for the early diagnosis and pharmacological treatment of CRC patients.

## Results

### *PEPT1* is repressed in CRC

**Fig. 1:**
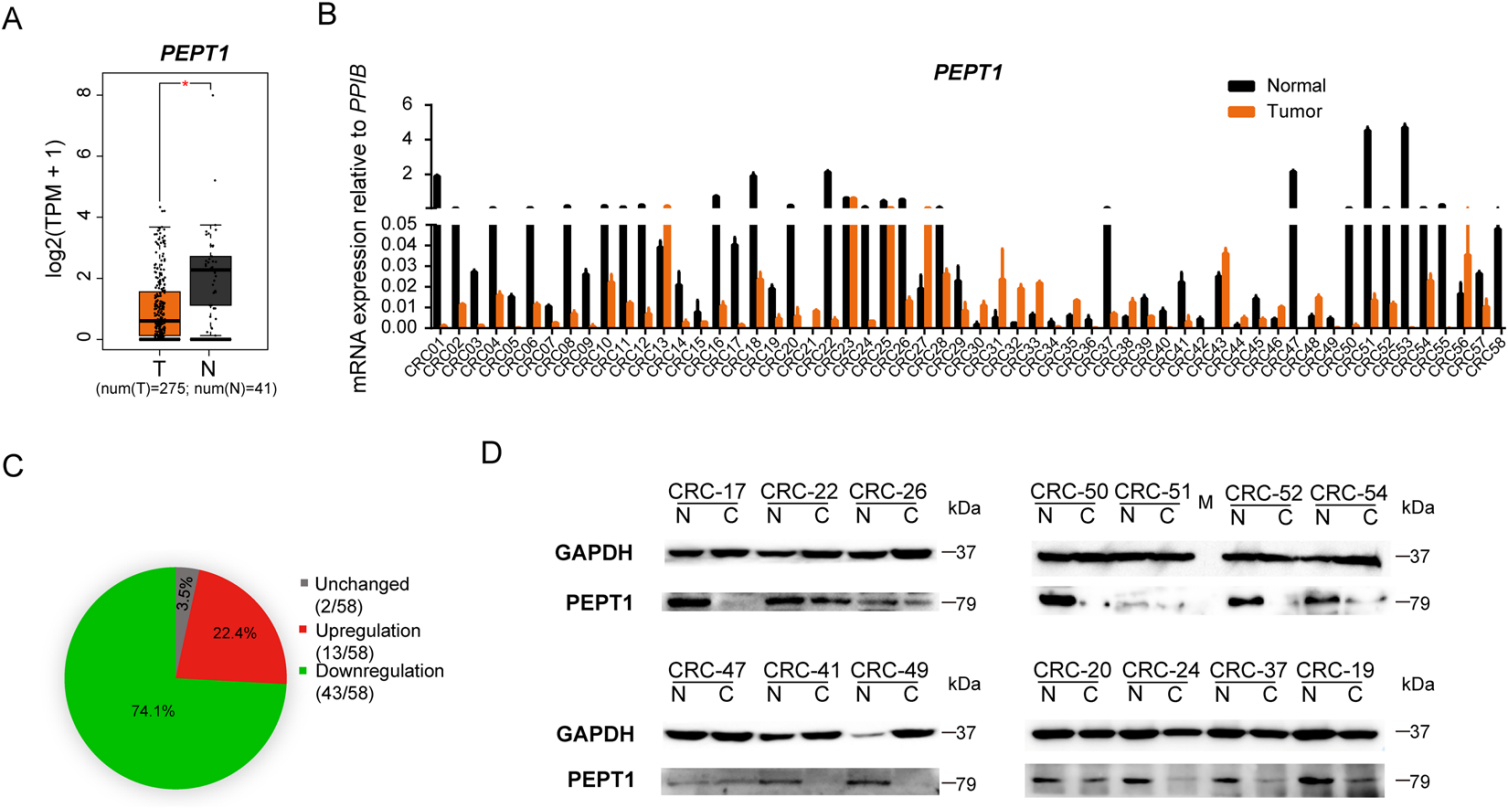
The mRNA and protein expression levels of PEPT1 are frequently downregulated in CRC. **A** *PEPT1* expression data were provided by TCGA in GEPIA. Box plots showed *PEPT1* mRNA expression in CRC tumor tissues (T) and normal tissues (N). *P < 0.05, one-tailed unpaired *t*-test. **B** The mRNA expression of *PEPT1* was detected in 58 pairs of CRC and corresponding adjacent non-tumorous tissues by RT-qPCR. Data are shown as means ± SD, *n* = 58. **C** The fold change log2(T/N) of PEPT1 expression between 58 pairs of CRC and adjacent normal tissues were summarized. <−1 is downregulation, −1 to 1 is unchanged and >1 is upregulation. **D** Representative images of western blotting analysis for the expression of PEPT1 in 14 paired samples of CRC. GAPDH was used as a loading control.

Boxplot analysis on GEPIA revealed that *PEPT1* transcription in CRC tumor tissues was markedly decreased compared to that in the normal colon (Fig. 1A). To confirm the mRNA expression levels of *PEPT1*, RT-qPCR was performed on 58 CRC tumor tissues and matched normal tissues (Fig. 1B, C). *PEPT1* expression was dramatically reduced in most CRC tissue samples (43/58). Next, we evaluated the protein expression of PEPT1 via western blotting (Fig. 1D). Thirteen of the 14 CRC patient samples displayed low PEPT1 expression (Fig. 1B), whereas 1 sample (CRC47) displayed no significant change in protein expression. Moreover, no significant correlation was found between PEPT1 expression levels and gender, age, TNM stage, location (Fig. S1A–D, Table S1). These data together demonstrate that PEPT1 is downregulated at both the mRNA and protein levels in most CRC tumors, regardless of gender, age, and TNM stage. These results imply that the expression of PEPT1 is an important factor for the diagnosis of CRC.

### DNMT1 mediates the suppression of *PEPT1*

**Fig. 2:**
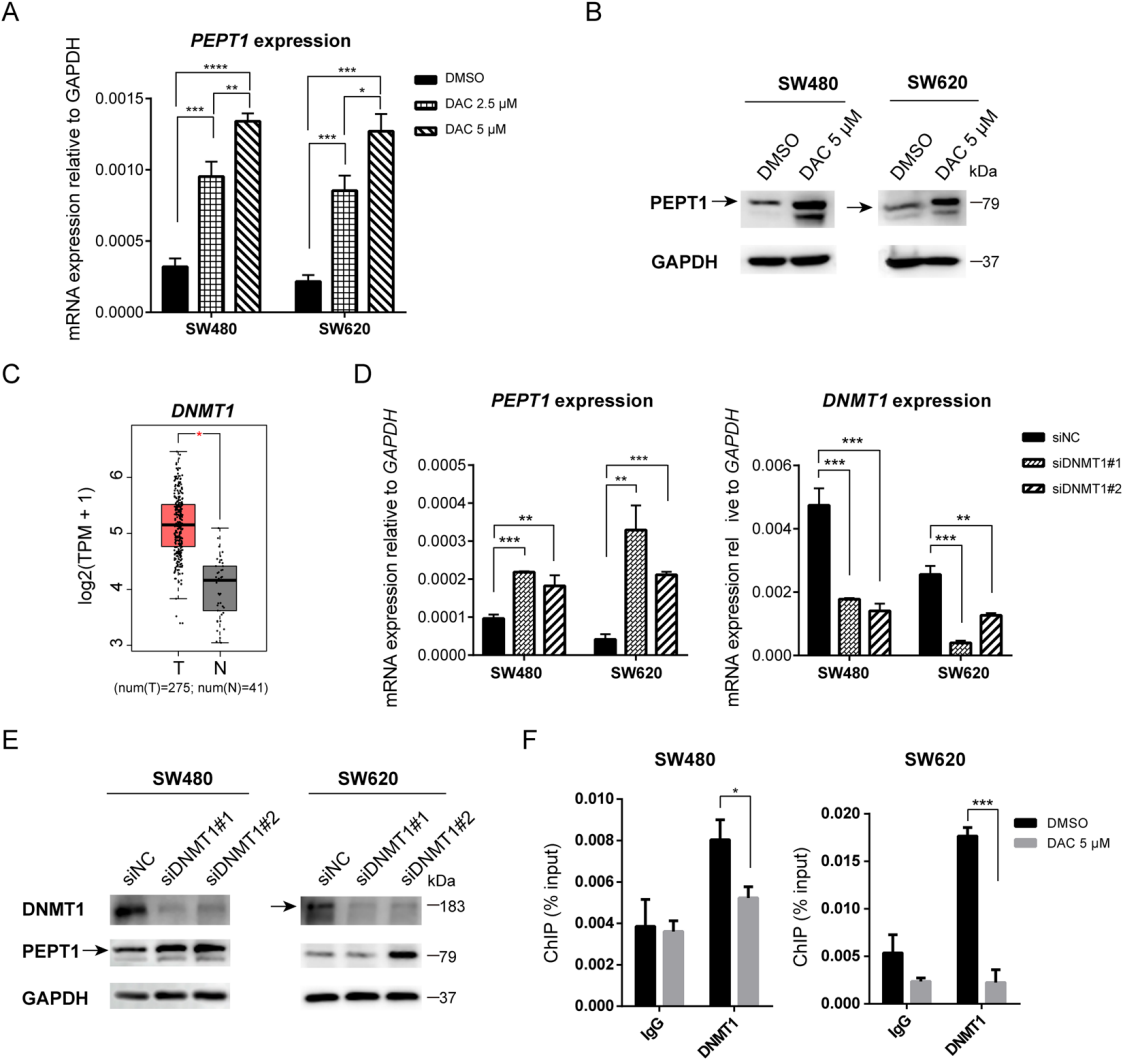
The inhibition of DNMT1 increases PEPT1 in CRC. **A** The mRNA expression of *PEPT1* in SW480 and SW620 cells. Cells were treated with DMSO, 2.5 μM DAC, or 5 μM DAC for 72 h. Data are shown as means ± SD, *n* = 3, two-tailed unpaired *t*-test, *P < 0.05, **P < 0.01, ***P < 0.001, ****P < 0.0001, significantly different from DMSO. **B** Immunoblotting confirmed DAC treatment in SW480 and SW620 cells. **C** *DNMT1* mRNA levels in CRC tumors (T) and normal tissues (N) from the TCGA in GEPIA. *P < 0.05, one-tailed unpaired *t*-test. **D** Knockdown of DNMT1 activated *PEPT1* mRNA expression in SW480 and SW620 cells, respectively. SiNC, cells transfected with negative control siRNA, siDNMT1#1, siDNMT1#2, two siRNAs for *DNMT1*. Data are shown as means ± SD, *n* = 3, two-tailed unpaired *t*-test, **P < 0.01, ***P < 0.001. **E** PEPT1 and DNMT1 protein expression after DNMT1 knockdown in SW480 and SW620 cells. **F** ChIP-qPCR analyses of DNMT1 enrichment at the proximal promoter of *PEPT1* in SW480 and SW620 cells after DAC (5 μM for 72 h) treatment. Data are shown as means ± SD, two-tailed unpaired t-test, *P < 0.05, ***P < 0.001.

The silencing of gene expression by CpG methylation is one of the most frequent epigenetic inactivation events. Therefore, we next investigated whether DNA methylation at the *PEPT1* promoter contributes to altered *PEPT1* transcription in CRC. As shown in Fig. 2A, we found that treatment with DAC, a demethylation reagent that blocks cellular DNA methyltransferases (DNMTs), activated *PEPT1* transcription in SW480 and SW620 cells in a dose-dependent manner. Western blot analysis confirmed that DAC induced PEPT1 expression at the protein level in both SW480 and SW620 cells (Fig. 2B). These data together imply that PEPT1 expression is controlled by DNA demethylation. As DNA methylation is catalyzed by DNMTs, including DNMT1, DNMT3a, and DNMT3b, we next investigated which DNMTs repressed *PEPT1* transcription in CRC. Among the three DNMTs, only DNMT1 was upregulated in CRC tissues compared with normal tissues (Figs. 2C, S2), which indicated that DNMT1 might be a crucial factor responsible for *PEPT1* repression. To investigate this further, we designed siRNAs against all 3 DNMTs to determine their role in regulating PEPT1. Interestingly, after transient transfection with siRNA, we found that PEPT1 mRNA and protein expression was upregulated by siDNMT1, but not siDNMT3a or siDNMT3b, in both SW480 and SW620 cells (Figs. 2D and E, S3). Furthermore, the ChIP results demonstrated that DNMT1 was absent at the *PEPT1* promoter after DAC treatment (Fig. 2F). As a control, DAC treatment showed no effect on the DNMT1 signature at the *GAPDH* promoter (Fig. S4A). Overall, our results indicate that DNMT1 is associated with transcriptional repression of *PEPT1* in CRC cells.

### DNA hypermethylation of *PEPT1*

**Fig. 3:**
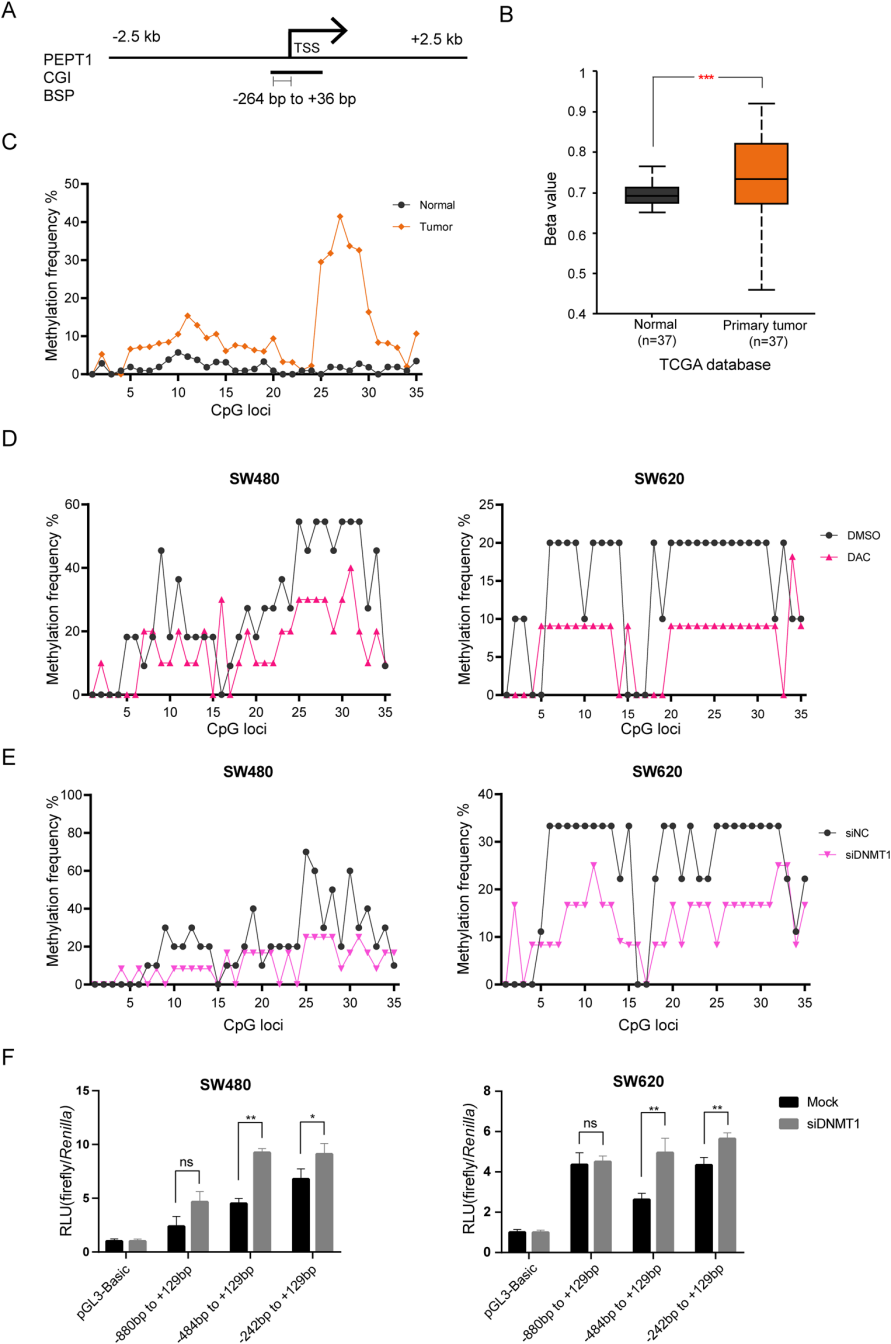
Repression of PEPT1 expression via DNMT1 mediated DNA hypermethylation. **A** *PEPT1* promoter region (±2.5 kb) around 5’ regions adjacent to the TSS. CGI, CpG islands. BSP, bisulfite-sequencing PCR. **B** DNA methylation data of *PEPT1* promoter in cancerous and adjacent non-tumorous tissues from the TCGA database (***P < 0.001). **C** BSP analysis of *PEPT1* CGI in cancerous and adjacent non-tumorous tissues (*n* = 11). Methylation percentages of the 35 CpG loci in the sequenced region were calculated. 11 pairs of CRC tissues belong to tissues in Fig. 1B (CRC17, 19, 22, 26, 41, 47, 49, 50, 51, 52, 54). The y axis indicates the average methylation percentage of each CpG site calculated from patient tissues with PEPT1 repression. **D**–**E** Calculation of methylation percentages of *PEPT1* promoter after DAC and siDNMT1 treatment in CRC cells. **F** Luciferase assay in DNMT1 knockdown CRC cells. Mock, cells transfected with negative control siRNA and *PEPT1* promoter constructs. Si-DNMT1, cells transfected with siDNMT1#2 and *PEPT1* promoter constructs. Data are shown as means ± SD, two-tailed unpaired *t*-test, *P < 0.05, **P < 0.01.

As shown in Fig. 3A, the upstream region around the TSS of *PEPT1* contains a putative CpG island (CGI, 900 bp). Using TCGA analysis, we found that promoter methylation of *PEPT1* is significantly increased in CRC tissues compared to normal tissues (Fig. 3B). The upstream CGI at the *PEPT1* proximal promoter region (−264 bp to +36 bp) contains 35 CpG sites. To directly determine whether the promoter region of *PEPT1* is subject to DNA hypermethylation, we next examined methylation status at the *PEPT1* promoter by BSP. To rule out unbiased amplification, the PCR reaction was optimized by combined bisulfite restriction analysis (Fig. S5). Eleven pairs of CRC and matched adjacent non-tumor tissues were used in the BSP. CRC tissues with the repression of *PEPT1* had higher levels of DNA methylation in the sequenced region (−264 bp to +36 bp) compared with adjacent non-tumor colonic tissue samples, especially in the 25th to 30th CpG sites, suggesting that hypermethylation occurs at the *PEPT1* promoter in CRC (Fig. 3C). Next, we examined whether exogenous DNA demethylation affects DNA methylation of the *PEPT1* promoter in CRC cells. The *PEPT1* promoter was hypermethylated in SW480 and SW620 cells (Fig. 3D). Upon treatment with DAC, globally inhibiting DNA methylation, CRC cells had decreased DNA methylation levels in the 25th to 30th CpG sites. In addition, a similar effect on the DNA methylation levels after DNMT1 siRNA transfection was also observed (Fig. 3E). As shown in Fig. 3F, knockdown of DNMT1 could also decrease the relative luciferase activity, indicating that DNMT1 binds directly to the promoter of *PEPT1*. Thus, we concluded that DNA hypermethylation mediated by DNMT1 represses *PEPT1* in CRC.

### Histone hypoacetylation represses *PEPT1* in CRC

**Fig. 4:**
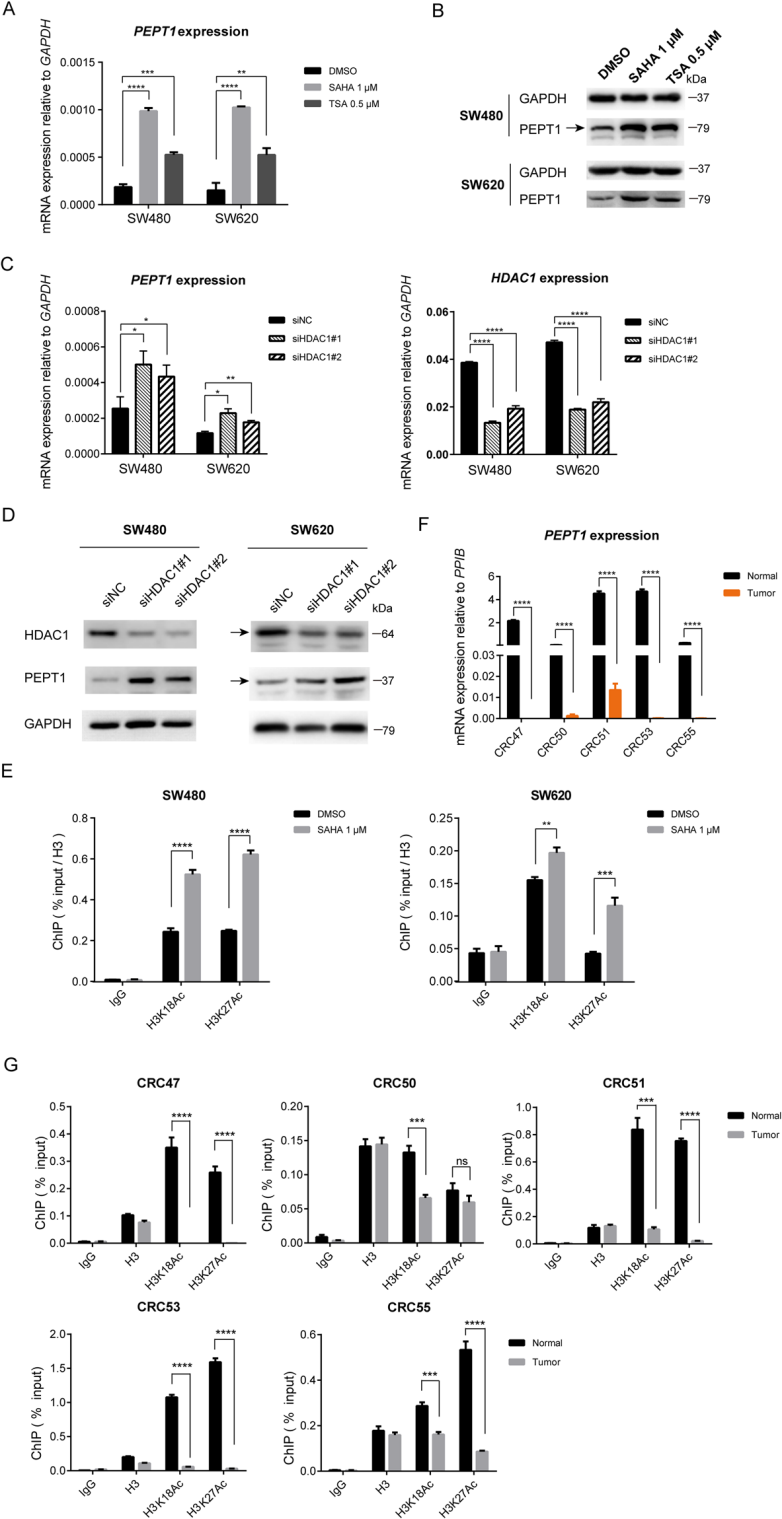
Histone hypoacetylation around *PEPT1* promoter in CRC. **A** The mRNA expression of *PEPT1* in SW480 and SW620 cells after HDAC inhibitors treatment. Cells were treated with DMSO, 1 μM SAHA for 48 h or 0.5 μM TSA for 24 h. Data are shown as means ± SD, *n* = 3, two-tailed unpaired *t*-test, ns, no significance, **P < 0.01, ***P < 0.001, ****P < 0.0001. **B** Immunoblotting confirmed HDAC inhibitors treatment in SW480 and SW620 cells. **C**–**D** Knockdown of HDAC1 activated PEPT1 mRNA and protein expression in SW480 and SW620 cells, respectively. SiNC, cells transfected with negative control siRNA, siHDAC1#1, siHDAC1#2, two siRNAs for *HDAC1*. Data are shown as means ± SD, *n* = 3, two-tailed unpaired *t*-test, *P < 0.05, **P < 0.01, ****P < 0.0001. **E** ChIP-qPCR analyses of H3K18/H3K27Ac occupancy at *PEPT1* promoter in SW480 and SW620 cells after SAHA (1 μM for 48 h) treatment. Data are shown as means ± SD, two-tailed unpaired *t*-test, **P < 0.01, ***P < 0.001, ****P < 0.0001. **F** *PEPT1* mRNA expression in CRC47, 50, 51, 53, 55. Data are shown as means ± SD, two-tailed paired *t*-test, **P < 0.01, ***P < 0.001. **G** ChIP-qPCR analyses of H3K18/K27Ac occupancy at the *PEPT1* promoter in five paired CRC tissues. Data are shown as means ± SD, two-tailed unpaired t-test, ***P < 0.001, ****P < 0.0001.

Histone acetylation is another important form of epigenetic regulation. We next attempted to decipher how histone acetylation contributes to *PEPT1* repression in CRC. We found that treatment with the HDAC inhibitors TSA and SAHA also strongly increased the mRNA and protein expression of *PEPT1* (Fig. 4A, B). Next, we treated cells with siRNAs targeting all HDAC family classes and observed significant upregulation of PEPT1 only after knockdown of HDAC1 (Figs. 4C and D, S3). These data suggest that hypoacetylation at the *PEPT1* promoter region caused by HDAC1 subsequently leads to the repression of *PEPT1* in CRC. In addition, ChIP-qPCR analyses demonstrated that the activating signals H3K18Ac and H3K27Ac increased after SAHA treatment (Figs. 4E, S4B). To confirm this result, we analyzed the occupancy of H3K18Ac and H3K27Ac to the promoter region of *PEPT1* in CRC samples. In five CRC tissues with repressed *PEPT1*, both H3K18Ac and H3K27Ac were decreased at the promoter region (Figs. 4F and G, S4C). Collectively, these results suggest that the absence of H3K18Ac and H3K27Ac combined with HDAC1-mediated deacetylation results in histone hypoacetylation at the *PEPT1* promoter region and transcriptional silencing of *PEPT1* in CRC.

### PEPT1 repression via the HDAC1-CBP/P300 axis

**Fig. 5:**
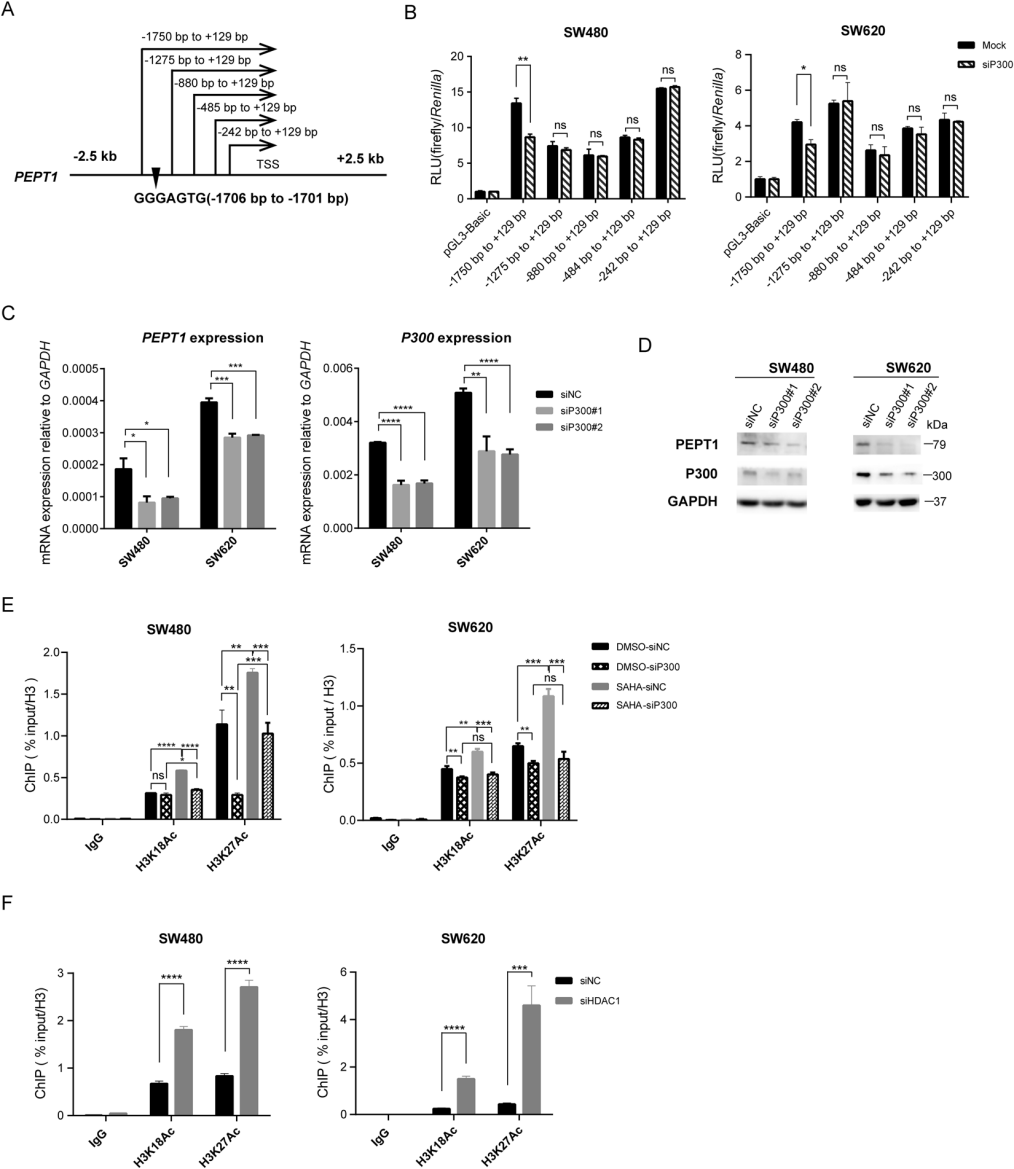
PEPT1 repression via HDAC1-CBP/P300 axis. **A** Promoter constructs of *PEPT1* in the luciferase assay. *GGGAGTG*, a consensus DNA binding sequence for p300. **B** Luciferase assay in P300 knockdown CRC cells. Mock cells transfected with negative control siRNA and promoter constructs. Si-P300, cells transfected with siP300#2 and promoter constructs. Data are shown as means ± SD, *n* = 3, two-tailed unpaired t-test, ns, no significance, *P < 0.05, **P < 0.01. **C**–**D** Inhibition of P300 downregulated PEPT1. SiNC, cells transfected with negative control siRNA, siP300#1, siP300#2, two siRNAs for *P300*. Data are shown as means ± SD, *n* = 3, two-tailed unpaired *t*-test, *P < 0.05, **P < 0.01, ***P < 0.001, ****P < 0.0001. **E** ChIP-qPCR analysis showed the effect of *P*300 expression on H3K18/K27Ac occupancy at the *PEPT1* promoter. Cells were treated with DMSO or 1 μM SAHA for 48 h. SiNC, cells transfected with negative control siRNA, siP300, cells transfected with siP300#2. Data are shown as means ± SD, two-tailed unpaired *t*-test, ns, no significance, **P < 0.01, ***P < 0.001, ****P < 0.0001. **F** ChIP-qPCR analysis showed the effect of HDAC1 expression on H3K18/K27Ac occupancy at the *PEPT1* promoter. SiNC, cells transfected with negative control siRNA, siHDAC1, cells transfected with siHDAC1#2. Data are shown as means±SD, two-tailed unpaired *t*-test, ***P < 0.001, ****P < 0.0001.

We next attempted to determine the role of positive and negative regulators of histone acetylation in regulating *PEPT1*. The acetyl group is usually added to lysine residues by histone acetyltransferases and removed by one of the HDAC enzymes. We performed dual-luciferase gene reporter assays to study whether histone acetyltransferases could bind to the UTR of *PEPT1* and regulate its expression. We transiently co-transfected CRC cells with five *PEPT1* promoter constructs based on the pGL3 luciferase reporter (Fig. 5A) and siRNAs targeting the histone acetyltransferases CBP/P300, which are specifically required for H3K18Ac and H3K27Ac^33,34^. We observed that inhibition of P300, but not CBP, reduced luciferase reporter activity by approximately 65% specifically at the region from −1750 to +129 bp in both SW480 and SW620 cells (Figs. 5B and S6). The luciferase assay results showed that P300 is a crucial factor for *PEPT1* regulation and that P300 trans-activation of the *PEPT1* promoter depends on the GGGAGTG sequence^35^, a consensus DNA-binding sequence for P300 (Fig. 5A, B). In addition, significantly lower expression levels of *PEPT1* were found after P300 depletion in SW480 cells. (Fig. 5C, D). ChIP analysis also revealed that, after SAHA treatment, H3K18Ac and H3K27Ac were unoccupied in P300-silenced SW480 cells but highly enriched in non-targeting siRNA-expressing cells (Figs. 5E, S4D). In addition, H3K18Ac and H3K27Ac around the *PEPT1* promoter were induced following knockdown of HDAC1 (Figs. 5F, S4E). These results together provide evidence that the HDAC1-CBP/P300 axis catalyzes H3K18Ac and H3K27Ac modification at the *PEPT1* promoter.

### Sensitization of CRC cells to UBEN by *PEPT1* activation

**Fig. 6:**
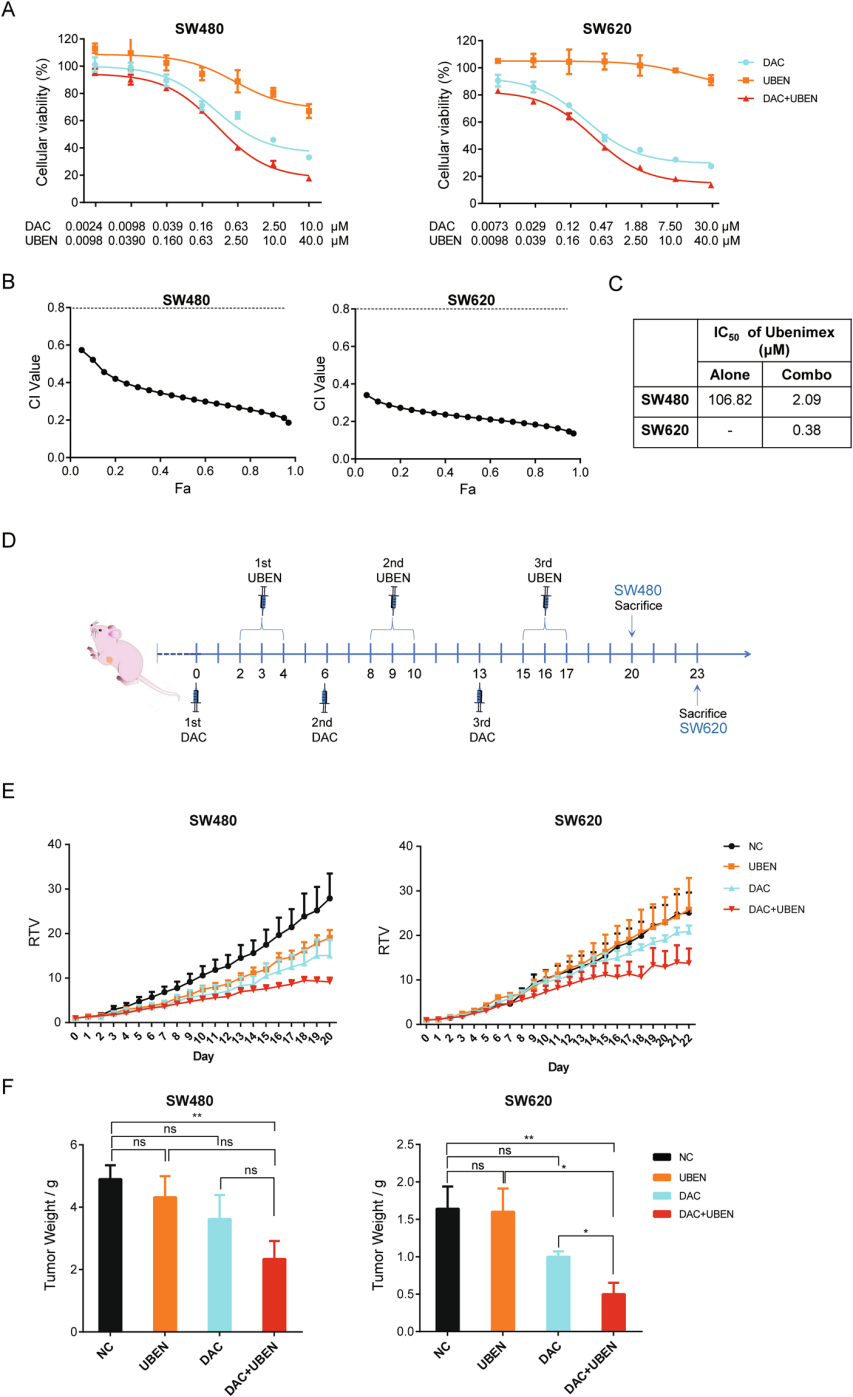
Activation of PEPT1 promotes the antitumor effects of UBEN in CRC. **A** Dose-effect curves of DAC, UBEN, and combination (DAC + UBEN) treatment in SW480 and SW620 cells. Cells were treated as indicated in Supplemental Table S2 and were subsequently analyzed using CCK8 assay. Data are shown as means ± SD, *n* = 6, nonlinear regression (curve fit) analysis. **B** Combination index (CI)–fraction affected (Fa) plots of DAC and UBEN combination were calculated by CompuSyn software in CRC cells. CI value is defined as follows: <0.8 is synergistic effect, from 0.8 to 1.2 is additive effect and >1.2 is antagonistic effect. **C** IC_50_ values of UBEN in CRC cells receiving combination treatment compared with UBEN alone. **D** Drug administration timeline and dosing schedule for xenograft models of CRC cells. **E** Relative tumor volume (RTV) curves in SW480 and SW620 xenograft models. Data represent the mean ± SD (*n* = 5). NC, UBEN, DAC, and DAC + UNEN indicate mice treated with sterile saline, ubenimex alone, decitabine alone, and decitabine-ubenimex combination, respectively. **F** Tumor weight of mice bearing SW480 and SW620 xenografts. Data represent means ± SD (*n* = 5), two-tailed unpaired *t*-test, ns, no significance, *P < 0.05, **P < 0.01.

UBEN, a dipeptide analog, has been demonstrated to have diverse antitumor and immunomodulatory effects^36,37^. As UBEN is a typical PEPT1 substrate and appears to be transported into cells predominantly by PEPT1^38,39^, the effects of UBEN are mediated by this oligopeptide transport activity^18^. Our earlier results showed that transcriptional repression of *PEPT1* was associated with DNA methylation and that the demethylation reagent DAC induced PEPT1 expression. We, therefore, examined whether a combination treatment of DAC and UBEN enhanced cell death relative to either agent alone in vitro and in vivo. As shown in Fig. 6A, DAC greatly enhanced the cytotoxic effect of UBEN. Based on the Chou-Talalay method^40^, the sequential combination of DAC and UBEN resulted in a synergistic effect, with a combination index (CI) value less than 0.8 (Fig. 6B). DAC pretreatment lowered the IC_50_ values for UBEN from 106.82 to 2.09 μM (51-fold improvement) in SW480, and from an undetermined IC_50_ to 0.38 μM in SW620 (Fig. 6C). This result gives a new insight into how understanding PEPT1 epigenetic regulation allows a targeted approach to the synergistic drug combination of DAC and UBEN. Finally, the in vivo effects of combination therapy with DAC and UBEN were analyzed in SW480 and SW620 xenograft models. The timeline of the three cycles of combination chemotherapy is shown in Fig. 6D. PEPT1 expression was induced in xenograft tumors 7 days after the first DAC pretreatment (Fig. S7A and B). The administration of DAC resulted in weak tumor-suppressive effects. However, the sequential combination of DAC and UBEN led to strong tumor suppression effects, with a 50% reduction in tumor weight (Figs. 6E, F and S7D). In contrast, there were no significant changes in body weight between the treated and untreated groups (Fig. S7C). Collectively, these results demonstrate that DAC treatment, which induces epigenetic alteration of *PEPT1*, enhanced cellular accumulation and increased cytotoxicity of UBEN both in vitro and in vivo.

## Discussion

UBEN, a CD13/aminopeptidase N inhibitor, has been used in adjuvant chemotherapy as an excellent anticancer immunopotentiator and has been found to have cytotoxic effects in several cancer-cell lines^41–44^. Previous studies reported that combining UBEN with anticancer drugs, such as 5-FU, CDDP, and DXR, can reverse the resistance of various cancer cells to anticancer drugs by increasing intracellular ROS levels^45^. However, further mechanisms or signaling pathways underlying resistance to UBEN have not been clearly elucidated. Cancer cells have been shown to defend themselves against some chemotherapeutics, at least in part, by repression of the transporters responsible for their uptake^4^. In this study, we attempted to reverse drug resistance by targeting the epigenetic mechanisms that alter the expression of the transporter responsible for the uptake of UBEN into cancer cells.

PEPT1 is increasingly recognized as an important determinant of drug efficacy and a promising and attractive target in prodrug design^15,46^. In this study, our present data suggesting that the mRNA and protein levels of PEPT1 are decreased in CRC. Importantly, we found that DAC induced PEPT1 and enhanced the cytotoxicity of UBEN against human CRC cells, suggesting that PEPT1 plays a crucial role in chemotherapy resistance. It is also possible that other peptide transporters, such as PHT1, PHT2, and in particular PEPT2, which has a structural resemblance to PEPT1, may contribute to multidrug resistance in CRC^47^. However, PEPT2 is not found in the intestine and is instead highly expressed in renal proximal tubular cells^47,48^. In addition, DAC did not alter the expression level of *PEPT2* in CRC cells (Fig. S8), indicating that UBEN is primarily transported by *PEPT1*, consequently resulting in high accumulation and increased cytotoxicity of UBEN after DAC treatment in CRC cells.

**Fig. 7:**
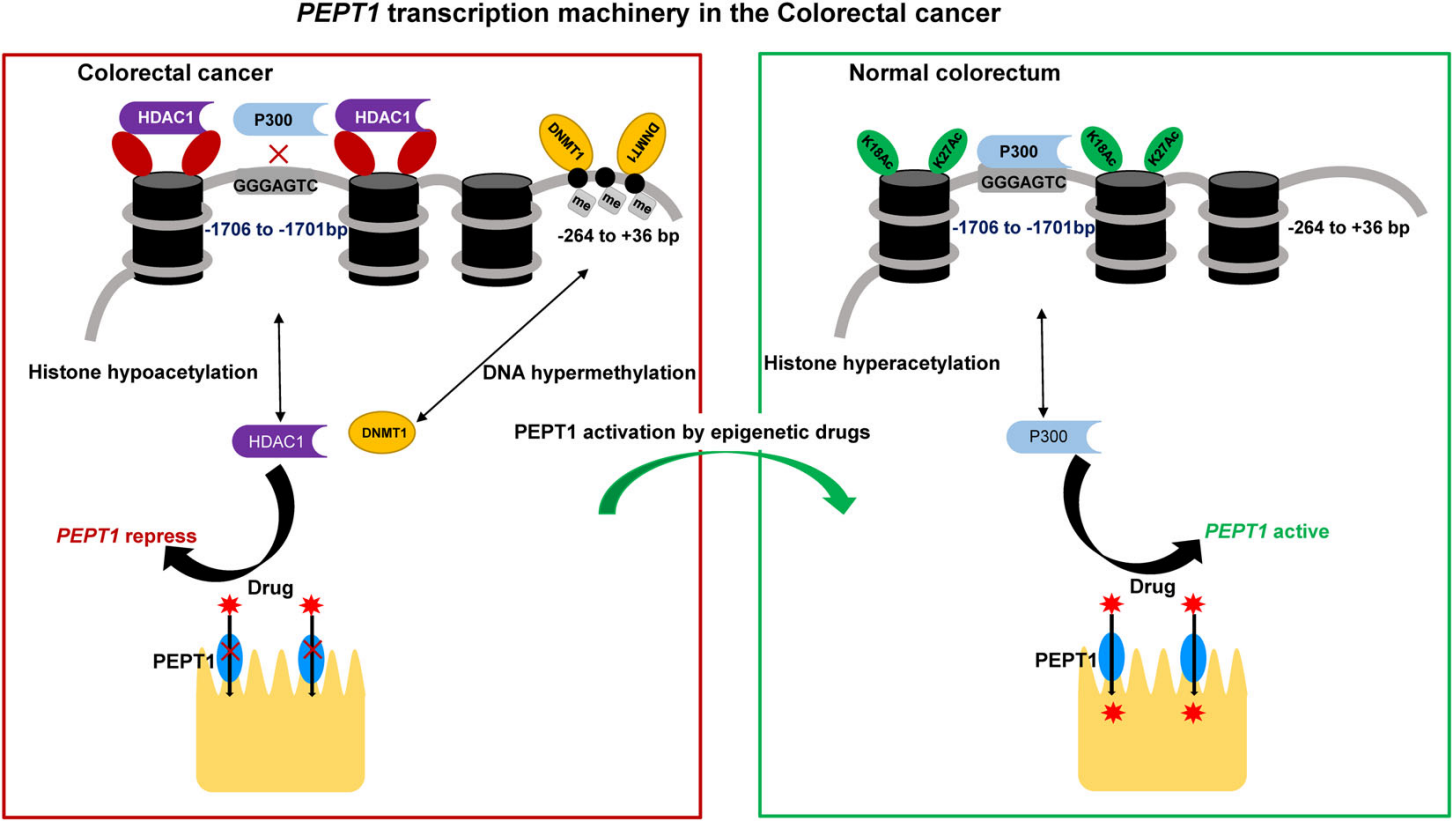
PEPT1 transcription machinery in Colorectal cancer. A hypomethylated CpG island at the proximal promoter and occupancy of H3K18/27Ac at distal promoters leads to transcriptional activation of PEPT1 in the normal colorectum. P300, but not CBP, is mainly responsible for H3K18/27ac around PEPT1 promoter and the element GGGAGTG (−1706 to 1701 bp) at PEPT1 promoter contributed to the basic transcription of PEPT1. Furthermore, the repressive PEPT1 promoter in CRC is characterized by a hypermethylated CpG island at the proximal promoter mediated by DNMT1 and the absence of H3K18/27Ac around the distal promoter due to HDAC1. The red star represents the dipeptide anti-cancer drug.

Our investigations into the epigenetic mechanisms underlying PEPT1 repression in CRC have indicated that both DNA methylation and histone acetylation regulate PEPT1 transcription (Fig. 7). We showed that a hypomethylated CpG island at the proximal promoter and occupancy of H3K18/27Ac at distal promoters leads to transcriptional activation of *PEPT1* in the normal colorectum. CREB-binding protein (CBP) and its paralog P300 are histone acetyltransferases capable of acetylating H3K18, H3K27, H3K56, H3K14, and H3K23^27,49^. Previous studies have demonstrated that CBP and P300 act as histone acetyltransferase complexes due to their conserved sequence regions^50,51^. Some evidence also indicates that CBP and P300 perform unique functions^52^. For example, P300, but not CBP, independent of its histone acetyltransferase activity, is required to induce Tripartite motif 22 in IFNγ-mediated antiviral activity^53^. In mouse embryonic stem cells, only P300, and not CBP, is a critical factor for maintaining H3K27Ac at specific promoter regions of the genome^54^. In contrast, CBP (but not P300 or PCAF) is responsible for the hyperacetylation of DDX21, which impairs its helicase activity and leads to the accumulation of R loops and DNA damage^55^. Consistent with this, our results indicate that P300 is predominantly responsible for H3K18/27ac around the *PEPT1* promoter. In addition, P300 contains an intrinsic and conserved DNA-binding domain, which shows a preferential affinity for the sequence GGGAGTG^35^. Our results confirmed that the element GGGAGTG (−1706 to 1701 bp) at the *PEPT1* promoter contributed to the transcription of *PEPT1*. Future studies on P300 catalytic core domains such as PHD fingers may further decipher the details of histone acetylation at the *PEPT1* promoter. Furthermore, we found that *PEPT1* promoter repression in CRC is associated with a hypermethylated CpG island at the proximal promoter mediated by DNMT1 and the absence of H3K18/27Ac around the distal promoter due to HDAC1. Aberrant DNMT1 expression has been detected in CRC^31,56^ and, consistently, our results showed that enrichment of DNMT1 at the proximal promoter region of *PEPT1* was responsible for *PEPT1* hypermethylation. In summary, our data suggest that alteration of DNA methylation, as well as histone acetylation, contribute to the repression of PEPT1, regardless of gender, age, TNM stage, and location in CRC. Promoter hyper-methylation is an early event in CRC carcinogenesis. Commercial Cologuard® Kit^57^, a multi-target stool DNA test that measures two methylation biomarkers (*BMP3*, *NDRG4*), is available for screening CRC. Aberrant DNA methylation at the proximal promoter region of the *PEPT1* gene (−264 to +36 bp) in stool, plasma, and serum might be beneficial for the diagnosis of CRC.

HDAC1-10 are essential transcriptional cofactors that lead to low acetylation. Interestingly, HDAC1 was also found to be overexpressed in CRC^58^ and promoted tumor angiogenesis by activating HIF1α/VEGFA^59^. A novel HDAC1 inhibitor, CBUD-1001, exerted anticancer effects by modulating apoptosis and EMT in CRC cells^60^. In our study, HDAC1 led to histone hypoacetylation at site H3K27, which was acetylated by P300. Moreover, DNMT1 itself was associated with deacetylase activity^61^, and when isolated from nuclear extracts, was found to be associated with HDAC1, as well as purified methyltransferase activity. It is therefore possible that the DNMT1/HDAC1 complex is required for maintaining the expression of *PEPT1*. Further research into the mechanisms of DNA methylation and its crosstalk with histone acetylation will be required in future studies.

## Conclusion

Thus, our study improves our understanding of the epigenetic repression of *PEPT1* promoters in CRC, we successfully applied this knowledge to design an epigenetic combination therapy sensitizing CRC to UBEN in vitro and in vivo.

## Materials and methods

### Patients

Fifty-eight fresh paired primary CRC specimens (Table S1) were prospectively collected for the study in one year. Of these, 14 paired CRC specimens (14/58) were obtained for the analysis of the PEPT1 protein level by western blotting. All tissue samples were obtained immediately after surgical resection from Hangzhou Cancer Hospital, with the patients’ written informed consent and approval from the Institutional Review Board of Hangzhou Cancer Hospital (Permit Number: HZCH-2017-09).

### Cell culture and drug treatment

The human CRC cell lines SW620 and SW480 were purchased from the cell bank of the Chinese Academy of Sciences (Shanghai, China, with STR detection ok) and were maintained in Leibovitz’s L-15 medium (Corning, USA) supplemented with 10% (v/v) fetal bovine serum (Gibco, USA) and 1% penicillin-streptomycin solution (New Cell & Molecular Biotech, China). Both cell lines were cultured at 37 °C in a humidified incubator without CO_2_ according to the instructions of the American Type Culture Collection (ATCC). UBEN was purchased from National Drug Reference Standards (purity 99.5%, Beijing, China). The DNMT inhibitor DAC and HDAC inhibitors trichostatin A (TSA) and SAHA (vorinostat) were purchased from Selleck (Houston, TX, USA) and dissolved in DMSO as a 50 mM stock solution. For drug treatment, cells at a confluency of 20–30% were treated with medium containing the indicated doses of DAC for 72 h, with fresh medium replacement every 24 h. Treatments with SAHA (48 h) and TSA (24 h) were performed similarly at cell densities of 40–50% and 60%, respectively.

### CCK8 assay

SW620 and SW480 cells were seeded into 96-well plates at a density of 1000 or 2000 cells per well, respectively, and pre-cultured overnight at 37 °C. In this study, we designed three groups of drug treatments, as listed in Table S2. For CCK8 assays, cells were treated with the drug treatments indicated for 72 h and subsequently maintained in a drug-free medium for 24 h. Next, 100 μL of fresh medium containing 10 µL CCK8 solution was added to each well of the 96-well plate, and the absorbance at 450 nm and 650 nm was read by a microplate reader (BioTek, USA). For drug synergy analysis, the combination index (CI) calculation was performed using Compusyn software (ComboSyn, Inc.) as described previously^40^. The CI values indicated the drug relationships as follows: <0.8 for synergism, 0.8 to 1.2 for additivity, and >1.2 for antagonism.

### Real-time quantitative polymerase chain reaction analysis

Total RNA was extracted from tissues and cells using the RNA mini-prep kit (Tiangen, China) and multisource total RNA mini-prep kit (Axygen, China), respectively. For gene expression studies, a reverse-transcription reaction was performed using PrimeScript RT Master Mix (Takara, Japan) to acquire cDNA, followed by a real-time quantitative polymerase chain reaction (RT-qPCR). The detection of amplification products conducted using TB Green Premix Ex Taq (Takara, Japan) in a 7500 Real-Time PCR instrument (Applied Biosystems). Relative gene expression values were calculated by the 2^−ΔΔCT^ method. All data were normalized to the reference genes *PPIB* and *GAPDH* for tissues and cell lines, respectively. The specific primers are listed in Table S3.

### Western blotting analysis

Whole-cell lysates of CRC tissues and cells were prepared in radioimmunoprecipitation assay (RIPA) buffer (Beyotime, China) supplemented with protease inhibitor cocktail (Beyotime, China), followed by centrifugation at 20,000×*g* for 10 min at 4 °C. The protein supernatants were collected and their concentration was quantified using a BCA Protein Assay Kit (Beyotime, China) to allow the dilution to similar concentrations with PBS and 5× protein loading buffer (Sangon, China). Samples were boiled for 10 min at 100 °C to allow denaturation and then SDS-PAGE analysis was performed immediately. Equal quantities of protein were loaded to each well and separated as follows: 70 V for 30 min in a 5% stacking gel, and then 150 V for 1.5 h in a 10% separating gel. The isolated protein was then transferred electrophoretically at 200 mA for 1.5 h from the gels onto PVDF membranes, followed by blocking in 5% skim milk at room temperature. Primary antibodies [anti-SLC15A1 (1:500, Abcam Cat# ab78020), anti-DNMT1 (1:1000, Abcam Cat# ab13537), anti-HDAC1 (1:2000, Abcam Cat# ab7028), anti-KAT3B/p300 (1;1000, Abcam Cat# ab14984), and anti-GAPDH (1:3000, Multi Sciences, Cat# Mab5465)] were incubated with PVDF membranes overnight at 4 °C. After washing, anti-rabbit IgG(H + L) and anti-mouse IgG(H + L) HRP-conjugated secondary antibodies (Multi-Science, China) were diluted 1:5000 and applied for 2 h at room temperature. Finally, the blots were washed three times with 1× TBST, and the immunoreactive protein bands were detected using ultra-sensitive ECL chemiluminescence substrate (4 A Biotech, China) in a C428-Odyssey-SA-GBOX Biosystem (LICOR, USA).

### Small interfering RNA transfection

The small interfering RNAs (siRNAs) for CBP/P300, DNMTs, and HDACs listed in Table S4 were synthesized by GenePharm (Shanghai, China) and transfected into CRC cells using Lipofectamine 3000 (Life Technologies, Waltham, MA) according to the manufacturer’s instructions. In brief, a mixture of Opti-MEM (Gibco, USA), 4 µl siRNA, and 5 µl Lipofectamine 3000 (Thermo Fisher, USA) was pre-incubated for 15 min at room temperature and then added to 70-80% confluent cells in a 6-well plate with fresh L-15 medium. A negative control siRNA was transiently transfected in the same way, and 48 h was the optimal sampling time for gene expression assays.

### Luciferase assays

The PGL3 Basic vector (Promega) was recombined with different sheared *PEPT1* promoter fragments to construct reporter plasmids. The promoter fragments of *PEPT1* were obtained by PCR with the specific primers listed in Table S3, with cell genomic DNA as an amplifying template. SW480 and SW620 CRC cells at 70–90% confluency in 24-well plates were transfected with siRNAs (as described in section 2.6) 24 h before plasmid transfection. After transfection for 48 h, cells were harvested and subjected to luciferase assay using the Dual-Luciferase Reporter Assay System (Promega, USA) according to the manufacturer’s protocol. The relative light unit (RLU) was calculated as the firefly luciferase activity from the promoter construct normalized to the corresponding Renilla luciferase activity of the same sample.

### Bisulfite sequencing analysis

Genomic DNA was extracted from CRC tissues and matched para-cancerous normal tissues, or CRC cell lines, using the QIAamp DNA Mini Kit (Qiagen, Germany). Sodium bisulfite conversion of genomic DNA and the subsequent cleanup of the converted DNA was carried out using the EpiTect Fast DNA Bisulfite Kit (Qiagen, Germany) according to the manufacturer’s instructions. The Bisulfite sequencing PCR (BSP) primers used were designed using the software MethPrimer^62^ and are listed in Table S3. The bisulfite-treated DNA was further amplified by PCR using Zymo Taq PreMix (Zymo Research, USA). PCR was performed under the following conditions: pre-denaturation at 95 °C for 10 min, followed by 40 cycles of denaturation at 95 °C for 30 s, annealing at 53 °C for 30 s, and extension at 72 °C for 1 min, followed by a final extension at 72 °C for 7 min. The annealing temperature used in the PCR reaction was optimized by combined bisulfite restriction analysis (COBRA)^63^ to eliminate biased amplification. In brief, the promoter region of PEPT1 (−2223 bp to +129 bp) was amplified and used as a reference template. The template was treated with (methylated) or without (mock-treated) CpG Methyltransferase M.SssI (New England Biolabs, USA) and S-adenosyl-L-methionine (SAM), followed by digestion with the methylation-sensitive restriction enzyme HpaII (New England Biolabs, USA) to identify the methylation efficiency. The methylated and mock-treated templates were converted and purified as described above. Subsequently, a 1:1 mixture of the fully methylated and mock-methylated samples was used as the template for gradient PCR. The PCR products were treated with MbiI (Thermo Fisher, USA), which only digested PCR products generated from methylated templates (as selected by Snake Charmer software). After restriction digestion, the DNA was analyzed using agarose gel electrophoresis. The annealing temperature at which both unmethylated and fully methylated DNA was amplified in an unbiased way was used in the BSP experiments. The purified PCR products were ligated with the pMD19-T vector using the DNA Ligation Kit Ver. 2.1 (Takara, Japan) and subsequently transformed into *E. coli* DH5α for sequencing. At least 10 clones for each sample were sequenced, and data analysis was performed using BiQ Analyzer.

### Chromatin immunoprecipitation (ChIP) assay

To further elucidate the epigenetic regulatory mechanisms of the target gene, we performed a ChIP assay adapted from that described previously^64^, with optimal modifications. The genomic DNA was broken into different-sized fragments, with 200–1000 bp DNA ladders interacting with histone acetylation marks, and 1000–2000 bp fragments being used for detection of transcription factor binding. CRC paired tissues and cells treated with drugs were crosslinked with PBS containing 1.1% formaldehyde (Thermo Forma, USA) on a rotating wheel before chromatin extraction, and then the chromatin was sheared by sonication. For tissue chromatin, an extra treatment with micrococcal nuclease (Sangong, China) was included to loosen chromatin before sonication. A 1% agarose gel was used to check the shearing efficiency. Proper sheared chromatin, together with ChIP-grade antibody and TrueBlot^®^ Anti-Rabbit Ig IP Agarose Beads (Rockland, USA), was incubated overnight at 4 °C with gentle shaking. After washing the antibody/chromatin/bead complexes with ice-cold LiCl washing buffer and TE buffer, the eluted antibody/chromatin in the supernatant was collected for reversion crosslinking in a 65 °C water bath overnight. Finally, following incubation with DNase-free RNase A (Tiangen, China) and proteinase K (Tiangen, China), the purified chromatin was extracted with phenol: chloroform: isoamyl alcohol (25:24:1, v/v) for subsequent RT-qPCR. Specific primers used in ChIP-qPCR are listed in Table S3. The enrichment of ChIP was indicated as the percentage (%) of input. ChIP-grade antibodies used in this study were as follows: anti-H3 (Abcam, Cat# ab1791), anti-H3Ac (Millipore, Cat# 06-599), anti-H3K9Ac (Abcam, Cat# ab4441), anti-H3K18Ac (Abcam, Cat# ab1191), anti-H3K27Ac (Abcam, Cat# ab4729), anti-DNMT1 (Abcam, Cat# 19905), and normal rabbit IgG (CST, Cat# 2729) as a negative control. Considering the difference in yield and purity between tumor tissues and adjacent tissues, as well as cells treated with drugs, the histone acetylation marks were normalized to the signal obtained with H3^65^.

### Animals and experimental design

Four-week-old female immune-deficient nude mice (BALB/c nude) were purchased from GemPharmatech (Jiangsu, China) and maintained under specific pathogen-free conditions with access to food and water *ad libitum* and under a constant temperature, humidity, and light cycle (12 h/12 h). All mouse experiments followed the relevant guidelines of Animal Welfare and were approved by the Zhejiang University Animal Care and Use Committee (Ethics Code: ZJU20200062). For experiments, 2.5 × 10^6^ SW620 or 1 × 10^7^ SW480 cells were suspended in 100 µL PBS and implanted subcutaneously into the right axilla of each mouse. When the tumor size approached 50 mm^3^, approximately one week later, mice were randomly divided into four treatment groups (*n* = 5): DAC (5 mg/kg b.w., i.p.), UBEN (15 mg/kg b.w., i.p.), combination (DAC and UBEN, i.p.), and negative control (blank solvent, sterile saline, i.p.), according to tumor volume. The doses of DAC and UBEN were based on a previous study^66^. The drug administration cycle comprised of three sequential DAC inductions on day 0, followed by 3-day UBEN treatment. The mouse weights and tumor sizes were measured and recorded every day. Tumor volumes were calculated using the formula *L* *×* *W*^2^*/2*, where *L* and *W* represent the longest and shortest dimensions, respectively. Relative tumor volume (RTV) and relative body weight (RBW) were normalized to the tumor volume and body weight on the first day of treatment with drugs, respectively. Mice were euthanized if the tumor size reached 2000 mm^3^ At the end of the experimental period (approximately 22 days), all animals were euthanized, and the primary xenograft tumors were extracted, weighed, and collected for further analysis.

### Statistical analysis

All results were expressed as the means ± SD and analyzed using GraphPad Prism 6 (GraphPad Software, USA). The statistical significance of two sets was calculated by unpaired *t*-test (one or two-tailed) as specifically mentioned, while the differences among groups were monitored by one-way analysis of variance (ANOVA), as per the significant level at *p* < 0.05. Drug IC50 calculations were performed using Prism version 6.0 with nonlinear regression (curve fit). Sample sizes of patient tissues and animal models were pre-calculated from our experience. Tissue samples were excluded from analysis when ∆Ct of housekeeping gene in paired tissues was more than 2. Animals were same-sex and age but were randomly divided into different groups. The investigator was not blinded to the group allocation during the experiment. Replicate of each experiment was listed in the figure legend.

## Supplementary information

Appendix A. Supplementary figures and Supplementary tables.

Appendix B. Original image files and descriptions of western blotting in figures.

## Data Availability

Chemical compounds studied in this article Decitabine (PubChem CID: 451668); Vorinostat (PubChem CID: 5311); Trichostatin A (PubChem CID: 444732); Ubenimex (PubChem CID: 72172). Key gene targets expressed in this article are hyperlinked to corresponding entries in the GEPIA2021 database (http://gepia2.cancerpku.cn/#analysis), which provides RNA sequencing (RNA-seq) data from 9736 tumors of The Cancer Genome Atlas (TCGA) database and 8587 normal samples of the Genotype-Tissue Expression (GTEx) database and offers tools for differential analysis, survival analysis, similar gene analysis, correlation analysis, and principal component analysis.
