# Supplementary material for: Epigenetic regulation of intestinal peptide transporter PEPT1 as a potential strategy for colorectal cancer sensitization
Source: Cell Death Dis. 2021 May 24;12(6):532. doi: 10.1038/s41419-021-03814-5 (PMC8144210; doi:10.1038/s41419-021-03814-5)
Supplement: Supplementary file 1 — Appendix A. Supplementary figures and Supplementary tables. [file 41419_2021_3814_MOESM1_ESM.docx]

**Appendix A. Supporting information**

**List of Supplementary Materials**

**Supplementary figures**

Figure S1. Association of *PEPT1* gene expression and patient information.

Figure S2. TCGA gene expression of DNMT3a and DNMT3b in CRC patients.

Figure S3. DNMTs and HDACs knockdown affect *PEPT1* expression in CRC cells.

Figure S4. ChIP-qPCR analysis at *GAPDH* promoters.

Figure S5. Bisulfite sequencing PCR was validated with control DNA template.

Figure S6. Luciferase assay in CBP knockdown CRC cells.

Figure S7. DAC induces PEPT1 expression in xenografted tumors and relative body weight curves in xenografts.

Figure S8. DAC treatment does not affect *PEPT2* expression on CRC cells.

**Supplementary tables**

Table S1. CRC tissue specimen information.

Table S2. DAC and UBEN combination schedule.

Table S3. Primers used in this study.

Table S4. SiRNAs used in this study.

**Supplementary Materials**

**
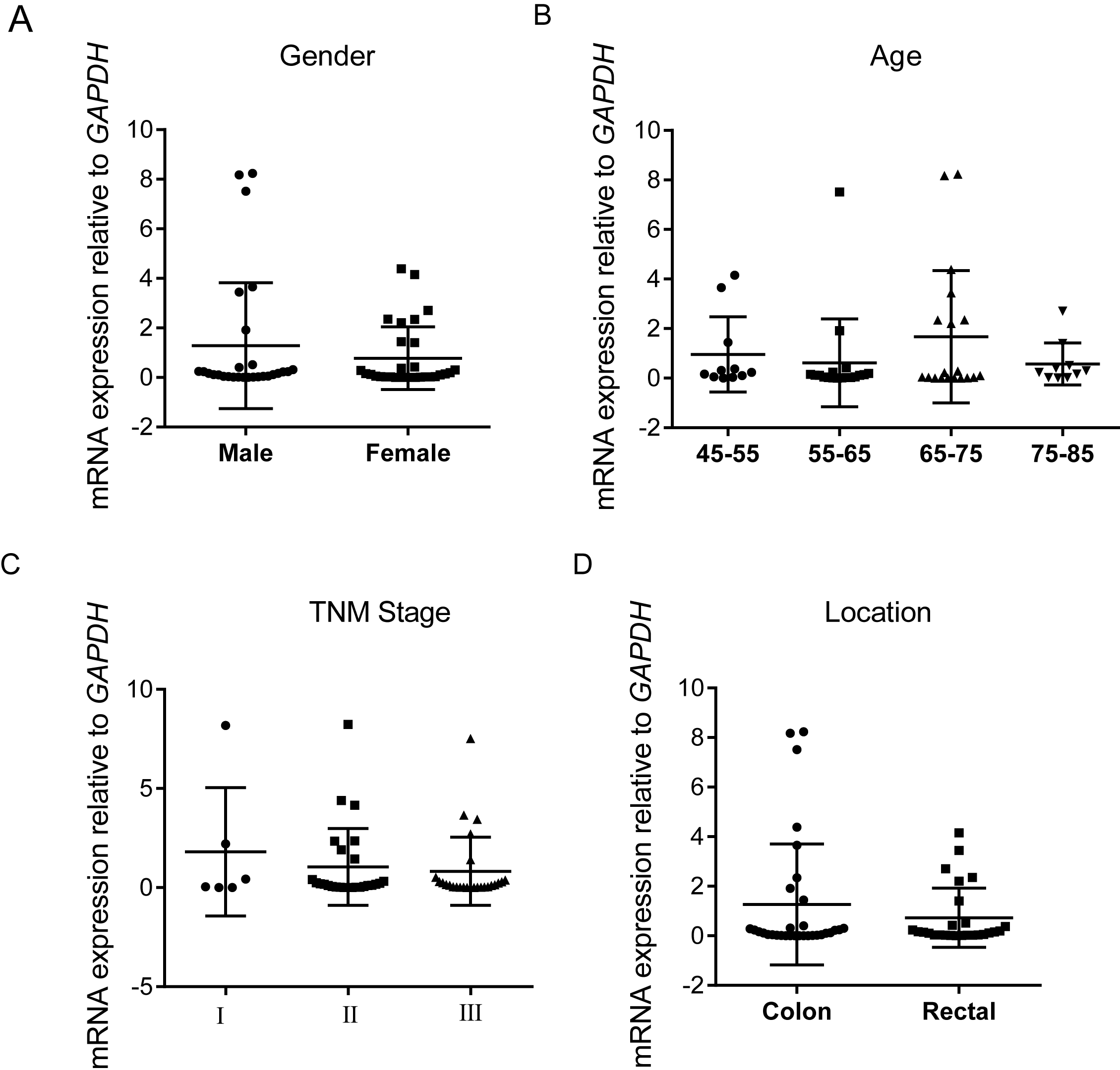
Figure S1.** **Association of *PEPT1* gene expression and patient information.** (A) Gender, unpaired t-test, P=0.3343. (B) Age, ANOVA analysis, P=0.3495. (C) TNM stage, ANOVA analysis, P=0.5660. (D) Location, unpaired t-test, P=0.3122.

**
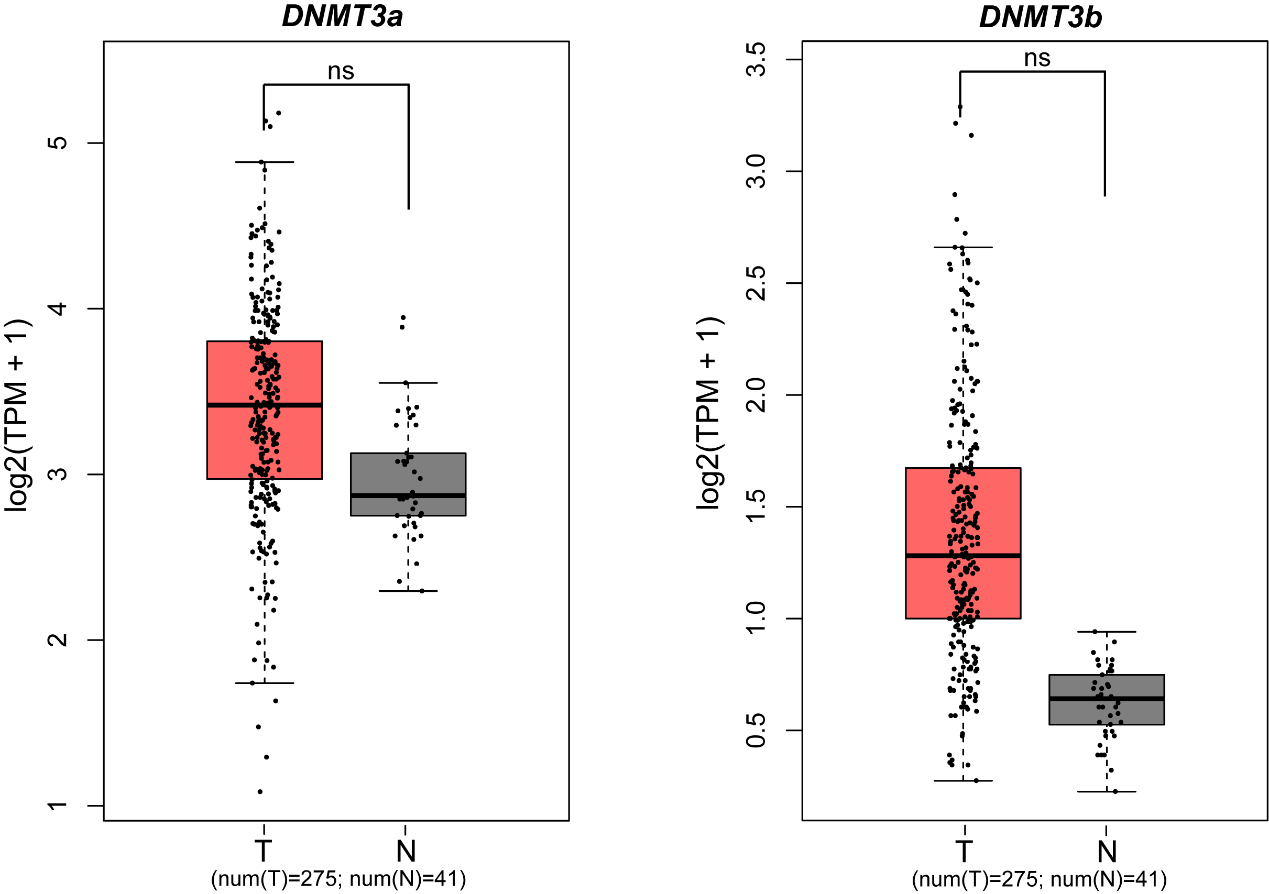
Figure S2. TCGA gene expression of DNMT3a and DNMT3b in CRC patients.** *DNMT3a* and *DNMT3b* mRNA levels in CRC tumors (T) and normal (N) tissues from the TCGA in GEPIA. Unpaired t-test. Ns, no significance.

**
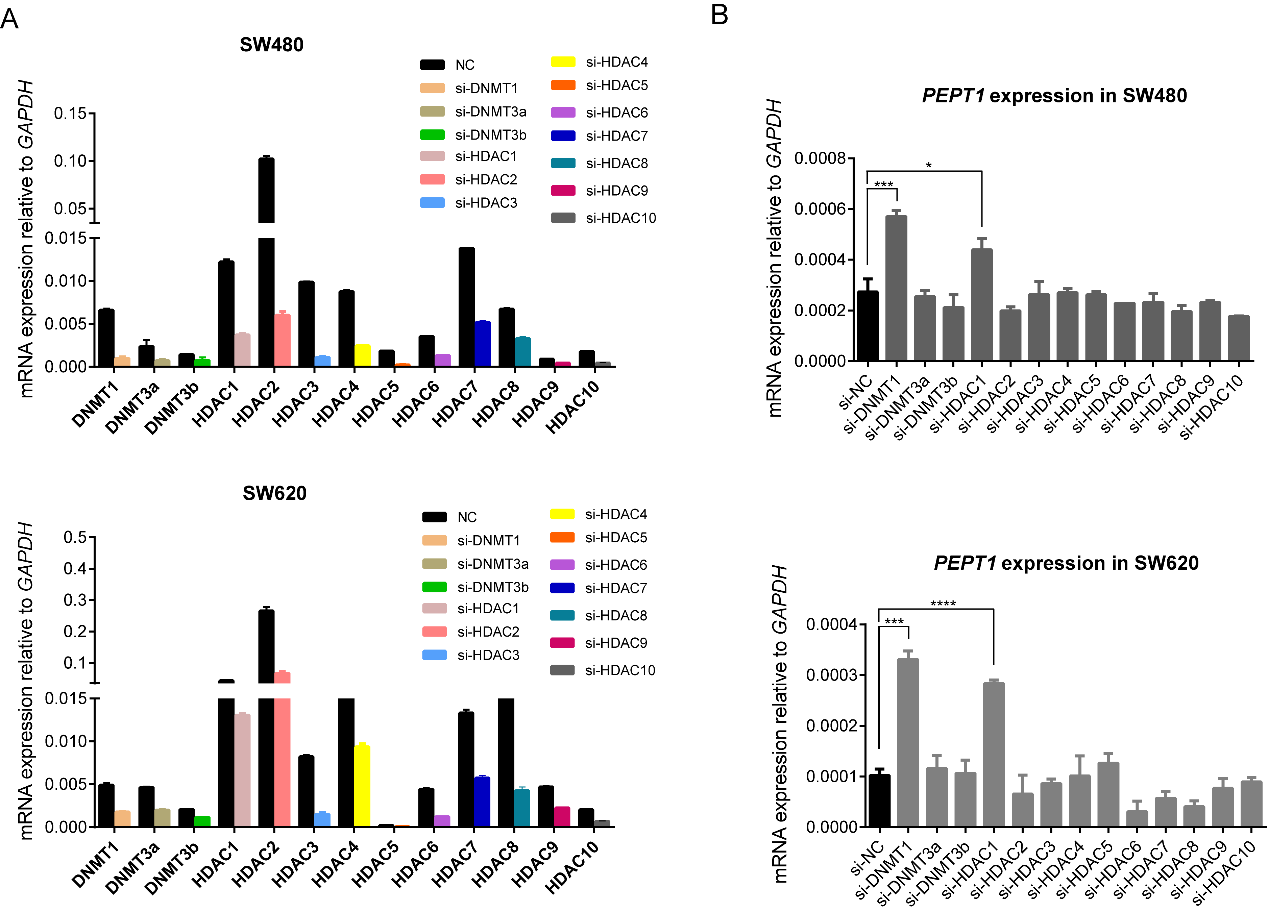
Figure S3.** **DNMTs and HDACs knockdown affect *PEPT1* expression in CRC cells.** (A-B) Inhibition of DNMTs and HDACs in CRC cells. The corresponding expression of *DNMTs* and HDACs (A), and *PEPT1* (B) after transfected by siDNMTs and siHDACs in CRC cells, respectively. Data are shown as means ± SD, two-tailed unpaired t-test, **P* < 0.5, ***P* < 0.01, ****P* < 0.001, *****P* < 0.0001.

**
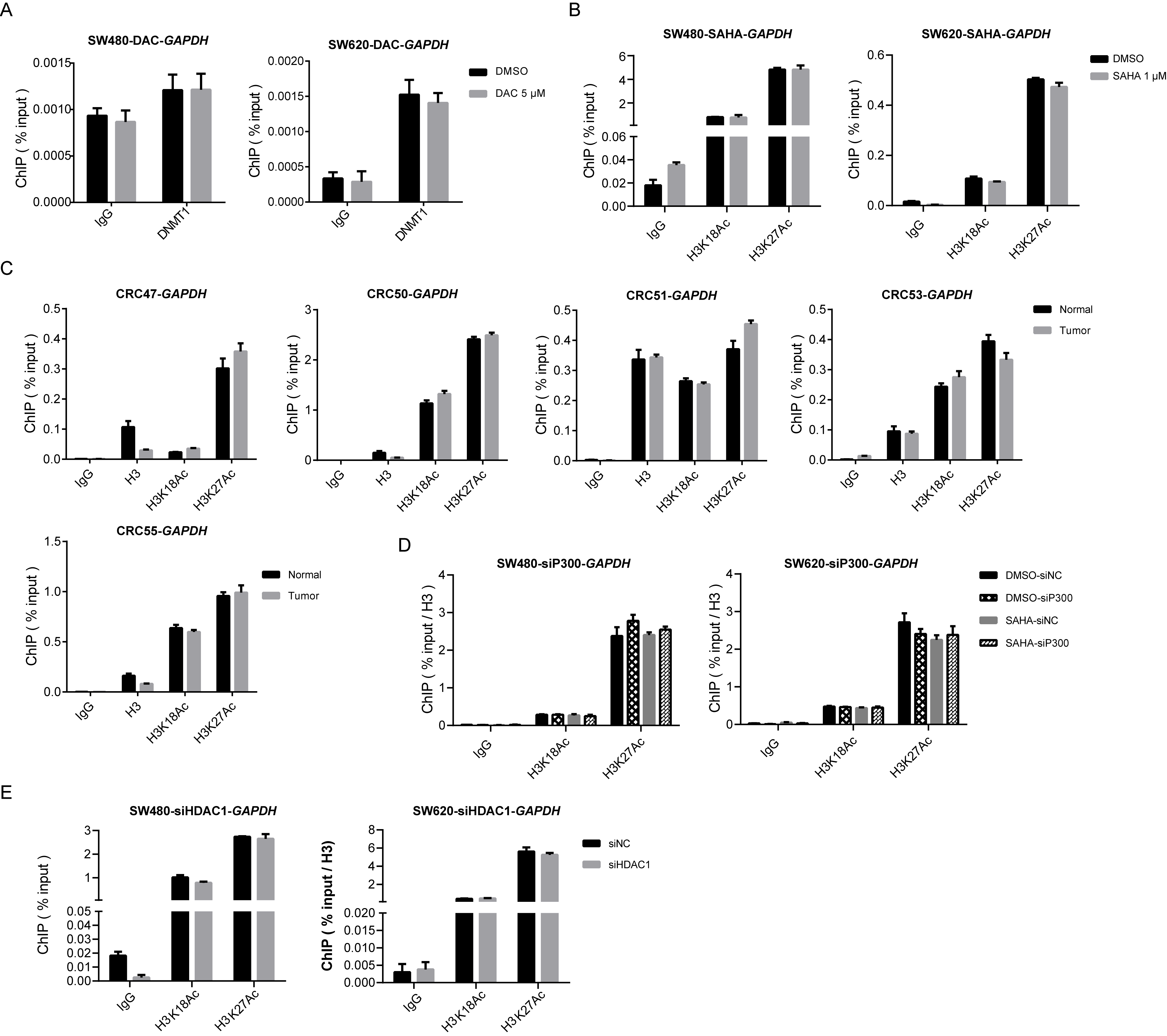
**

**Figure S4. ChIP-qPCR analysis at *GAPDH* promoters.** (A) ChIP-qPCR analyses of DNMT1 enrichment at the proximal promoter of *GAPDH* in SW480 and SW620 cells after DAC (5μM for 72 hours) treatment. (B) ChIP-qPCR analyses of H3K18/H3K27Ac occupancy at *GAPDH* promoter in SW480 and SW620 cells after SAHA (1μM for 48 hours) treatment. (C) ChIP-qPCR analyses of H3K18/K27Ac occupancy at the *GAPDH* promoter in five paired CRC tissues. (D) ChIP-qPCR analysis showed the effect of P300 expression on H3K18/K27Ac occupancy at the *GAPDH* promoter. (E) ChIP-qPCR analysis showed the effect of HDAC1 expression on H3K18/K27Ac occupancy at the *GAPDH* promoter. Data are shown as means ± SD, two-tailed unpaired t-test, ns, P>0.05.

**
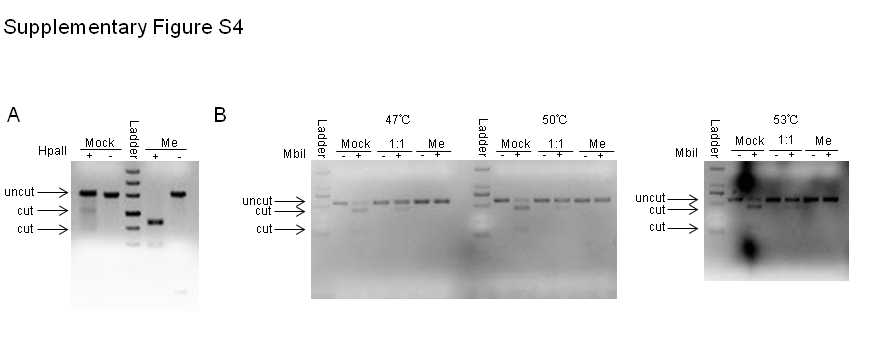
****Figure S5.** **Bisulfite sequencing PCR was validated with control DNA template.** Validation of Bisulfite sequencing PCR assay using in vitro methylated DNA (A) Control DNA templates (-2223 bp to +129 bp of PEPT1 promoter) were methylated in vitro by M.SssI (Me) or mock-treated (Mock) and then were digested with methylation-sensitive HpaII restriction enzymes, which is presumed to digest only non-methylated CCGG sequence. The restriction digest of DNA was analyzed by 1% agarose gel electrophoresis. (B) Determine suitable annealing temperatures by combined bisulfite restriction analysis (COBRA). Three different annealing temperatures were indicated. The bisulfite converted DNA mixture (Me: Mock = 1:1) served as a PCR template and were digested with methylation-sensitive Mbil after amplification. The digested DNA was analyzed by agarose gel electrophoresis. 53℃ was the optimal temperature for unbiased amplification.

**
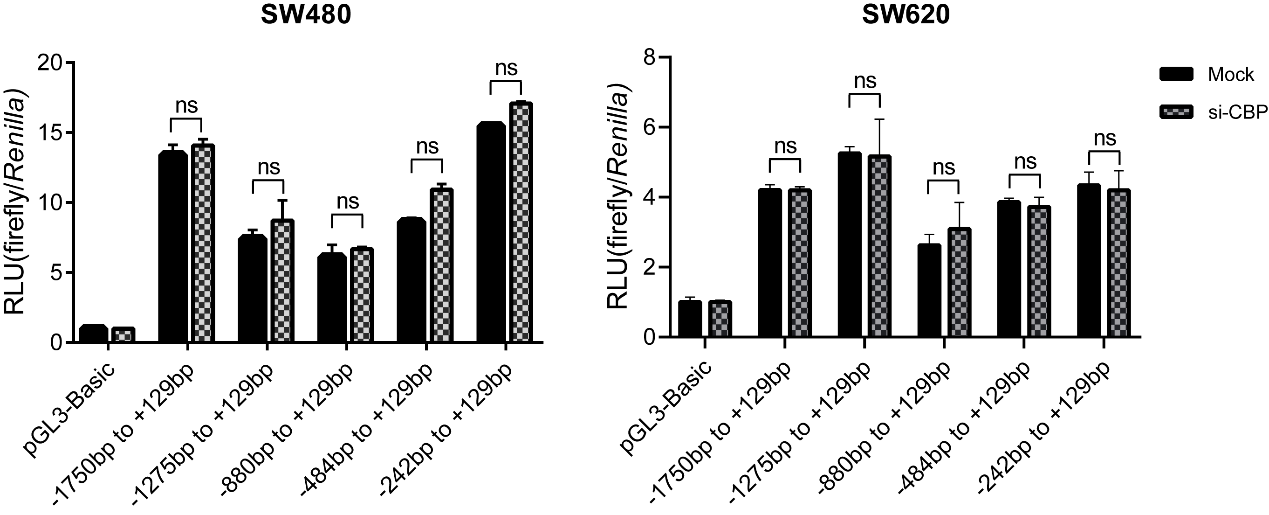
Figure S6.** **Luciferase assay in CBP knockdown CRC cells.** Mock, cells transfected with negative control siRNA and promoter constructs. Si-CBP, cells transfected with siCBP and promoter constructs. Data are shown as means ± SD, two-tailed unpaired t-test, ns, no significant.

**
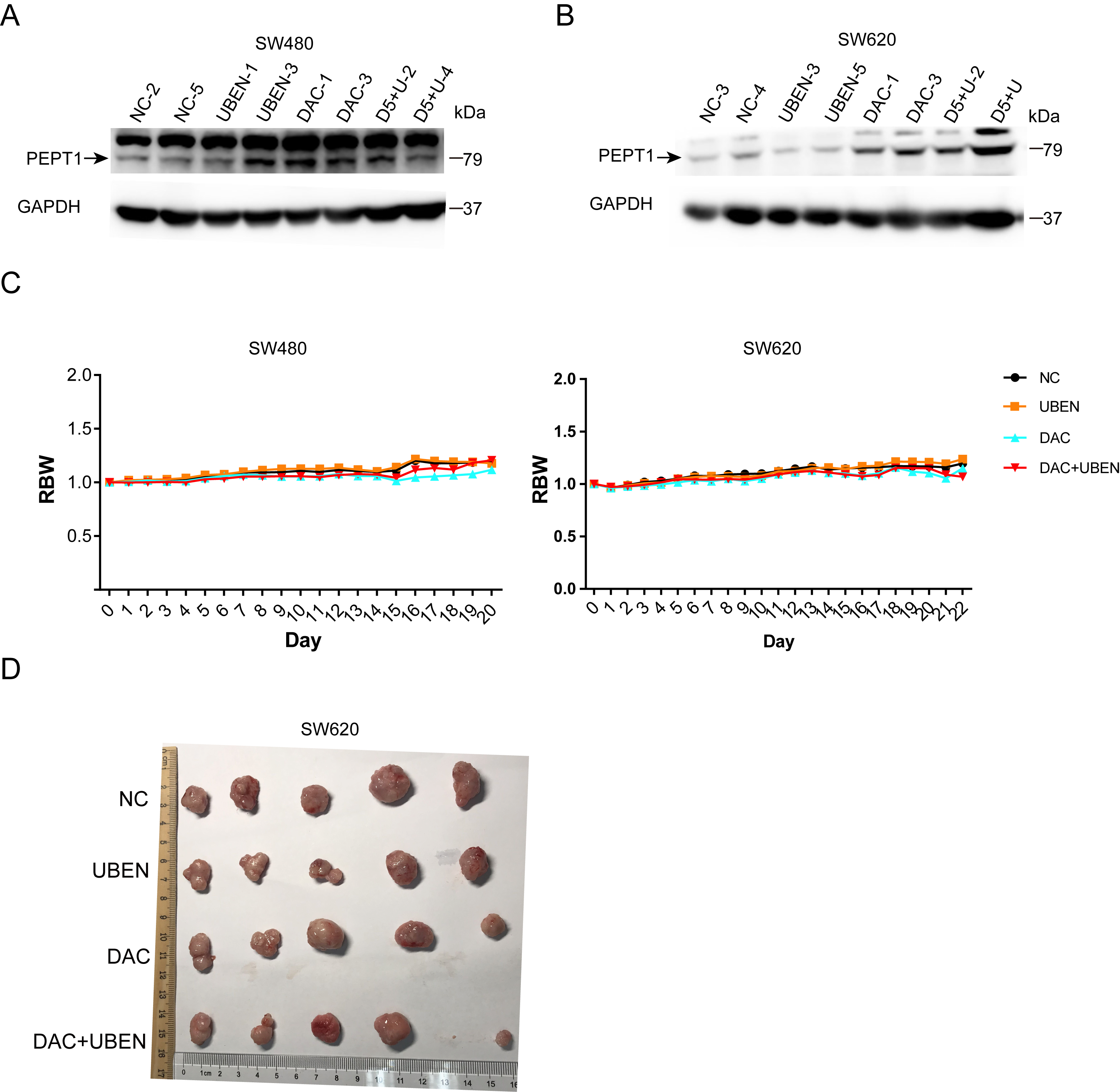
Figure S7. DAC induces PEPT1 expression in xenografted tumors and relative body weight curves in xenografts.** (A-B) DAC induces PEPT1 expression in SW480 and SW620 xenografted tumors. (C) Relative body weight (RBW) curves in SW480 and SW620 xenograft models. Data represent the mean ± SD (n=5). NC, UBEN, DAC, and DAC+UNEN indicate mice treated with sterile saline (NC), ubenimex alone (UBEN), decitabine alone (DAC), and decitabine-ubenimex combination (DAC+UBEN), respectively. (D) The size distribution of primary tumors after resection from each group of SW620 xenografts at day 23.

**
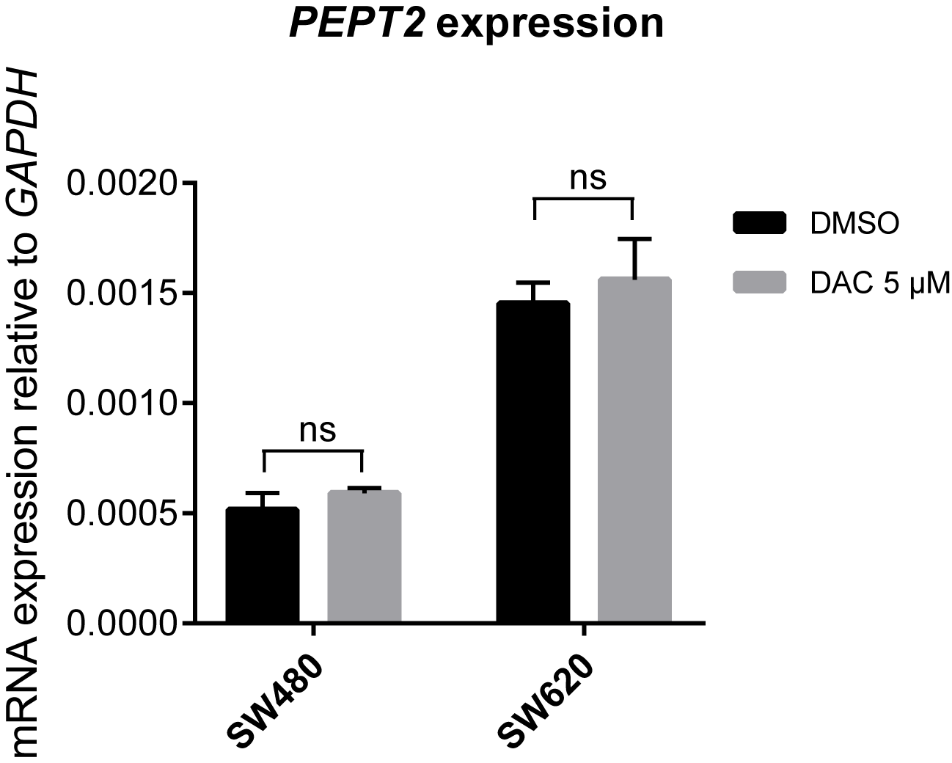
**

**Figure S8. DAC treatment does not affect *PEPT2* expression on CRC cells.** The mRNA expression of *PEPT2* in SW480 and SW620 cells after DAC treatment for 72 hours. Data are shown as means ± SD, two-tailed unpaired t-test, ns, no significant.

**Supplementary Table S1.** CRC tissue specimen information.

| Number | Gender | Age | Subtype | TNM stage |
| --- | --- | --- | --- | --- |
| 1 | Male | 71 | rectal adenocarcinoma | T2N0M0 |
| 2 | Male | 65 | rectal adenocarcinoma | T3N0M0 |
| 3 | Female | 49 | colon adenocarcinoma | T3N0M0 |
| 4 | Female | 78 | rectal adenocarcinoma | T3N1M0 |
| 5 | Female | 63 | rectal adenocarcinoma | T3N0M0 |
| 6 | Male | 56 | rectal adenocarcinoma | T3N1M0 |
| 7 | Female | 52 | colon adenocarcinoma | T2N1M0 |
| 8 | Female | 68 | colon adenocarcinoma | T2N0M0 |
| 9 | Female | 61 | colon adenocarcinoma | T3N1M0 |
| 10 | Female | 59 | rectal adenocarcinoma | T3N0M0 |
| 11 | Male | 50 | rectal adenocarcinoma | T3N0M0 |
| 12 | Male | 53 | rectal adenocarcinoma | T3N0M0 |
| 13 | Male | 55 | rectal adenocarcinoma | T3N0M0 |
| 14 | Male | 59 | rectal adenocarcinoma | T3N2aM0 |
| 15 | Male | 76 | rectal adenocarcinoma | T3N1M0 |
| 16 | Female | 60 | colon adenocarcinoma | T3N1M0 |
| 17 | Male | 69 | rectal adenocarcinoma | T2N1M0 |
| 18 | Male | 82 | rectal adenocarcinoma | T3N0M0 |
| 19 | Female | 64 | colon adenocarcinoma | T3N0M0 |
| 20 | Male | 81 | colon adenocarcinoma | T3N1M0 |
| 21 | Female | 63 | colon adenocarcinoma | T3N0M0 |
| 22 | Male | 74 | colon adenocarcinoma | T3N1M0 |
| 23 | Male | 73 | colon adenocarcinoma | T3N1M0 |
| 24 | Female | 70 | rectal adenocarcinoma | T2N1M0 |
| 25 | Female | 47 | rectal adenocarcinoma | T3N0M0 |
| 26 | Male | 82 | rectal adenocarcinoma | T4N0M0 |
| 27 | Female | 46 | colon adenocarcinoma | T3N1M0 |
| 28 | Female | 80 | rectal adenocarcinoma | T3N2M0 |
| 29 | Male | 56 | rectal adenocarcinoma | T2N0M0 |
| 30 | Female | 70 | colon adenocarcinoma | T3N0M0 |
| 31 | Female | 69 | colon adenocarcinoma | T2N0M0 |
| 32 | Female | 59 | colon adenocarcinoma | T3N1M0 |
| 33 | Female | 68 | rectal adenocarcinoma | T2N1M0 |
| 34 | Female | 68 | colon adenocarcinoma | T3N0M0 |
| 35 | Male | 74 | rectal adenocarcinoma | T2N0M0 |
| 36 | Male | 58 | rectal adenocarcinoma | T3N0M0 |
| 37 | Male | 61 | colon adenocarcinoma | T3N2M0 |
| 38 | Male | 68 | colon adenocarcinoma | T3N0M0 |
| 39 | Female | 83 | colon adenocarcinoma | T3N0M0 |
| 40 | Female | 72 | colon adenocarcinoma | T3N0M0 |
| 41 | Female | 79 | colon adenocarcinoma | T3N0M0 |
| 42 | Female | 56 | colon adenocarcinoma | T3N1M0 |
| 43 | Male | 34 | colon adenocarcinoma | T3N0M0 |
| 44 | Male | 73 | colon adenocarcinoma | T3N0M0 |
| 45 | Male | 77 | colon adenocarcinoma | T3N1M0 |
| 46 | Male | 67 | rectal adenocarcinoma | T3N0M0 |
| 47 | Male | 69 | colon adenocarcinoma | T4N0M0 |
| 48 | Male | 78 | rectal adenocarcinoma | T3N1M0 |
| 49 | Female | 64 | colon adenocarcinoma | T3N1M0 |
| 50 | Male | 61 | rectal adenocarcinoma | T3N1M0 |
| 51 | Male | 49 | colon adenocarcinoma | T3N0M0 |
| 52 | Male | 52 | rectal adenocarcinoma | T3N1M0 |
| 53 | Male | 69 | colon adenocarcinoma | T2N0M0 |
| 54 | Female | 53 | colon adenocarcinoma | T3N0M0 |
| 55 | Female | 72 | colon adenocarcinoma | T2N1M0 |
| 56 | Female | 68 | colon adenocarcinoma | T3N1M0 |
| 57 | Male | 64 | rectal adenocarcinoma | T3N1M0 |
| 58 | Female | 58 | colon adenocarcinoma | T3N0M0 |

**Supplementary Table S2.** DAC and UBEN Combinations.

|  | DRUG TREATMENT | | | |
| --- | --- | --- | --- | --- |
|  | 0 hour | 24 hours | 48 hours | 72 hours |
| DAC | DAC | DAC | DAC+DMSO | DMSO |
| UBEN | DMSO | DMSO | UBEN+DMSO | DMSO |
| DAC+UBEN | DMSO | DMSO | DAC+UBEN | UBEN |

**Supplementary Table S3.** Primers used in this study.

| **Application** | **Gene** | **Primer (5′–3′)** |
| --- | --- | --- |
| RT-qPCR | PEPT1 | TCCACCGCCATCTACCATAC |
|  |  | GGACAAACACAATCAGGGCT |
|  | DNMT1 | CAACGAGTCTGGCTTTGAGA |
|  |  | GACACAGGTGACCGTGCTTA |
|  | DNMT3a | CGTTGGCATCCACTGTGA |
|  |  | AATGGTCCTCACTTTGCTGAA |
|  | DNMT3b | AGAGGGACATCTCACGGTTC |
|  |  | GGTTGCCCCAGAAGTATCG |
|  | GAPDH | AGGTGAAGGTCGGAGTCA |
|  |  | GGTCATTGATGGCAACAA |
|  | PPIB | TGTGGTGTTTGGCAAAGTTC |
|  |  | GTTTATCCCGGCTGTCTGTC |
|  | HDAC1 | ACGAAGACGACCCTGACAAG |
|  |  | TCCTCACAGGCAATTCGTTT |
|  | HDAC2 | ATAAAGCCACTGCCGAAGAA |
|  |  | TCCTCCAGCCCAATTAACAG |
|  | HDAC3 | ACGTGGGCAACTTCCACTAC |
|  |  | GACTCTTGGTGAAGCCTTGC |
|  | HDAC4 | AAGAACAAGGAGAAGGGCAAG |
|  |  | TGGAGAACTCTGGTCAAGGGA |
|  | HDAC5 | GTCTCGGCTCTGCTCAGTGTAGA |
|  |  | GGCCACTGCGTTGATGTTG |
|  | HDAC6 | AGTCTTATGGATGGCTATTGCATG |
|  |  | TGGACCAGTTAGAGGCCTTCAGG |
|  | HDAC7 | CCATGACGACGGCAACTTCTT |
|  |  | TGCTGCGTCATGTATCCAAAAC |
|  | HDAC8 | AAGAGGGCGATGATGATC |
|  |  | GTGGCTGGGCAGTCATAACC |
|  | HDAC9 | AGTGTGAGACGCAGACGCTTAG |
|  |  | TTTGCTGTCGCATTTGTTCTTT |
|  | HDAC10 | TTACTTCTCCTGGCACCGCTA |
|  |  | CCACGTAGTCAGCGTTTCCC |
|  | CBP | TTTCCGGCAGCCTGTAGAT |
|  |  | TCCATGGGATTCTTTACGATG |
|  | P300 | TTCAGCACCATGGACAGTTG |
|  |  | GTTGCATACGAGGCCCATAG |
| Promoter constructs | PEPT1  -1750 to +129bp | ACGCGTTGAGGAATTGCCACACTGTCT |
|  |  | CTCGAGGTAGCTCCGAGTCTTTAGCCC |
|  | PEPT1  -1275 to +129bp | ACGCGTTTGCCATTGCTCTTGGTGTT |
|  |  | CTCGAGGTAGCTCCGAGTCTTTAGCCC |
|  | PEPT1  -880 to +129bp | ACGCGTAGCATGATGCCTCCAGCTTT |
|  |  | CTCGAGGTAGCTCCGAGTCTTTAGCCC |
|  | PEPT1  -485 to +129bp | ACGCGTCACATGCTCACACTCAATCCT |
|  |  | CTCGAGGTAGCTCCGAGTCTTTAGCCC |
|  | PEPT1  -242 to +129bp | ACGCGTCCCCGACCTCCTGAGTCA |
|  |  | CTCGAGGTAGCTCCGAGTCTTTAGCCC |
| BSP | PEPT1-Promoter  -2223 to +129bp | ACGCGTTGCTGCACCCATCAACTCAT |
|  |  | CTCGAGGTAGCTCCGAGTCTTTAGCCC |
|  | PEPT1-B4 | GTAGAGTTGGGGTTGTATTTGG |
|  |  | CTCCTCTTAAACCAAAATTCCCTAC |
| ChIP-qPCR | PEPT1-ch2 | TGCCTTAGTTCCAGGGTGAT |
|  |  | CGTCAGCAAAACAGCTAGGA |
|  | GAPDH-ch4 | GAAGGGCTTCGTATGACTGG |
|  |  | CTTAAGGCATGGCTGCAACT |

Restriction enzyme sites in construct primers are shown in red.

**Supplemental Table S4.** siRNAs used in this study.

| Name | Sense (5′–3′) |
| --- | --- |
| siNC | UUCUCCGAACGUGUCACGU |
| siDNMT1#1 | GGAGAACGGUGCUCAUGCUU |
| siDNMT1#2 | GCCCAAUGAGACUGACAUCAA |
| siDNMT3a | GGGUUGGACAUCAUCUCCU |
| siDNMT3b | AGAUGACGGAUGCCUAGAG |
| siHDAC1#1 | CCGGUCAUGUCCAAAGUAA |
| siHDAC1#2 | CGGUUAGGUUGCUUCAAUCUA |
| siHDAC2 | CUACGACGGUGAUAUUGGA |
| siHDAC3 | AAAGCGAUGUGGAGAUUUA |
| siHDAC4 | GCAAGAUCCUCAUCGUGGA |
| siHDAC5 | ACACGUUCAUGCUAAAGCA |
| siHDAC6 | CCGUGAGAGUUCCAACUUU |
| siHDAC7 | UCACUGACCUCGCCUUCAA |
| siHDAC8 | GGACGGUACUACAGUGUAA |
| siHDAC9 | GAAAGACACUCCAACUAAU |
| siHDAC10 | GGUGAACAGUGGUAUAGCA |
| siP300#1 | AAGUUCAAACGCCGAGUCUUC |
| siP300#2 | GCACGAACUAGGAAAGAAA |
| siCBP | GCAAACAGAGCAUGGUCAA |
