# Supplementary material for: Epigenetic regulation of intestinal peptide transporter PEPT1 as a potential strategy for colorectal cancer sensitization
Source: Cell Death Dis. 2021 May 24;12(6):532. doi: 10.1038/s41419-021-03814-5 (PMC8144210; doi:10.1038/s41419-021-03814-5)
Supplement: Supplementary file 2 — Appendix B. Original image files and descriptions of western blotting in figures. [file 41419_2021_3814_MOESM2_ESM.pdf]

Figure1D

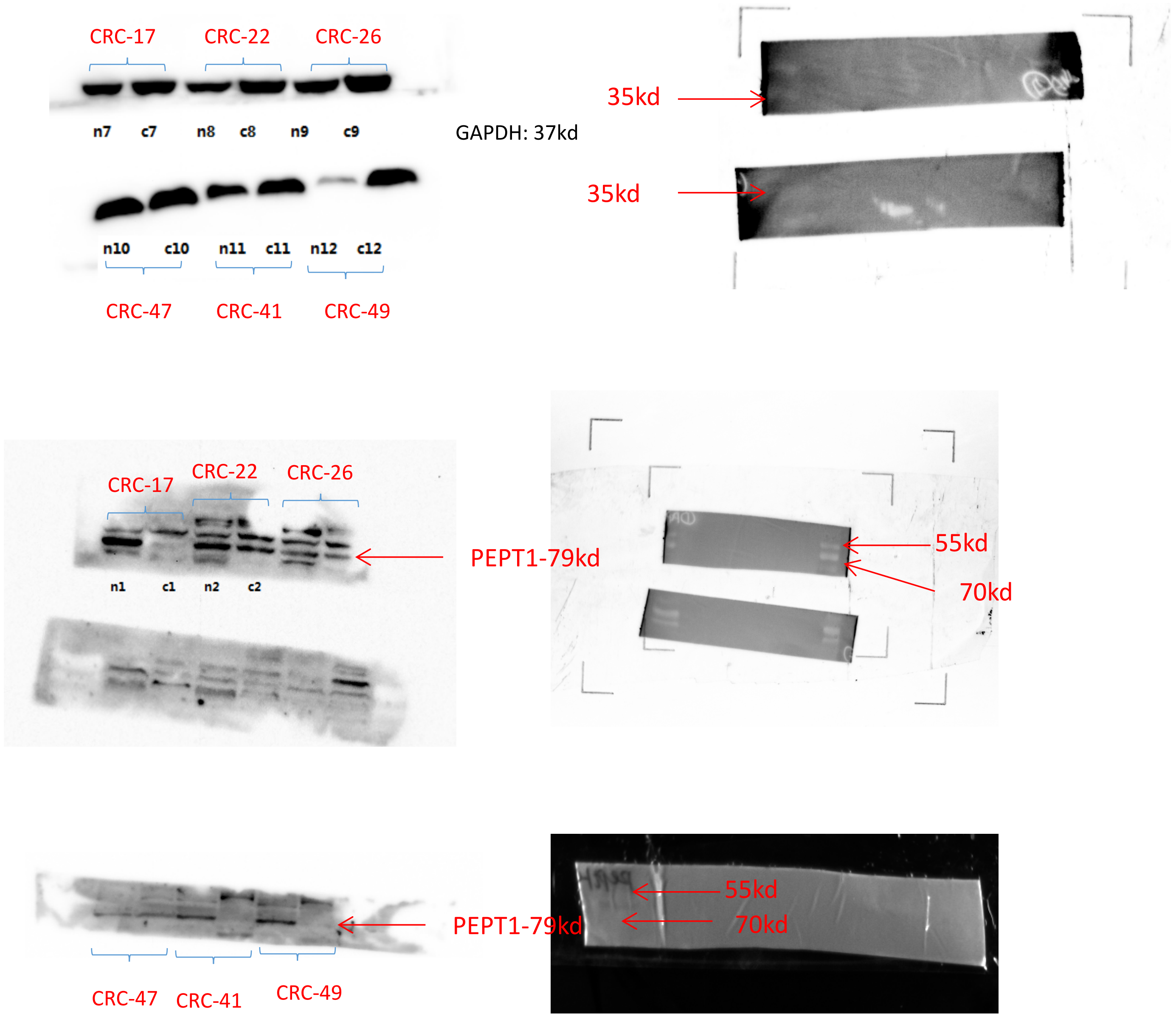

CRC-17
CRC-22
CRC-26
n7 c7 n8 c8 n9 c9
GAPDH: 37kd
35kd
35kd
n10 c10 n11 c11 n12 c12
CRC-47
CRC-41
CRC-49
CRC-17
CRC-22
CRC-26
PEPT1-79kd
n1 c1 n2 c2
55kd
70kd
PEPT1-79kd
55kd
70kd
CRC-47
CRC-41
CRC-49

Figure1D

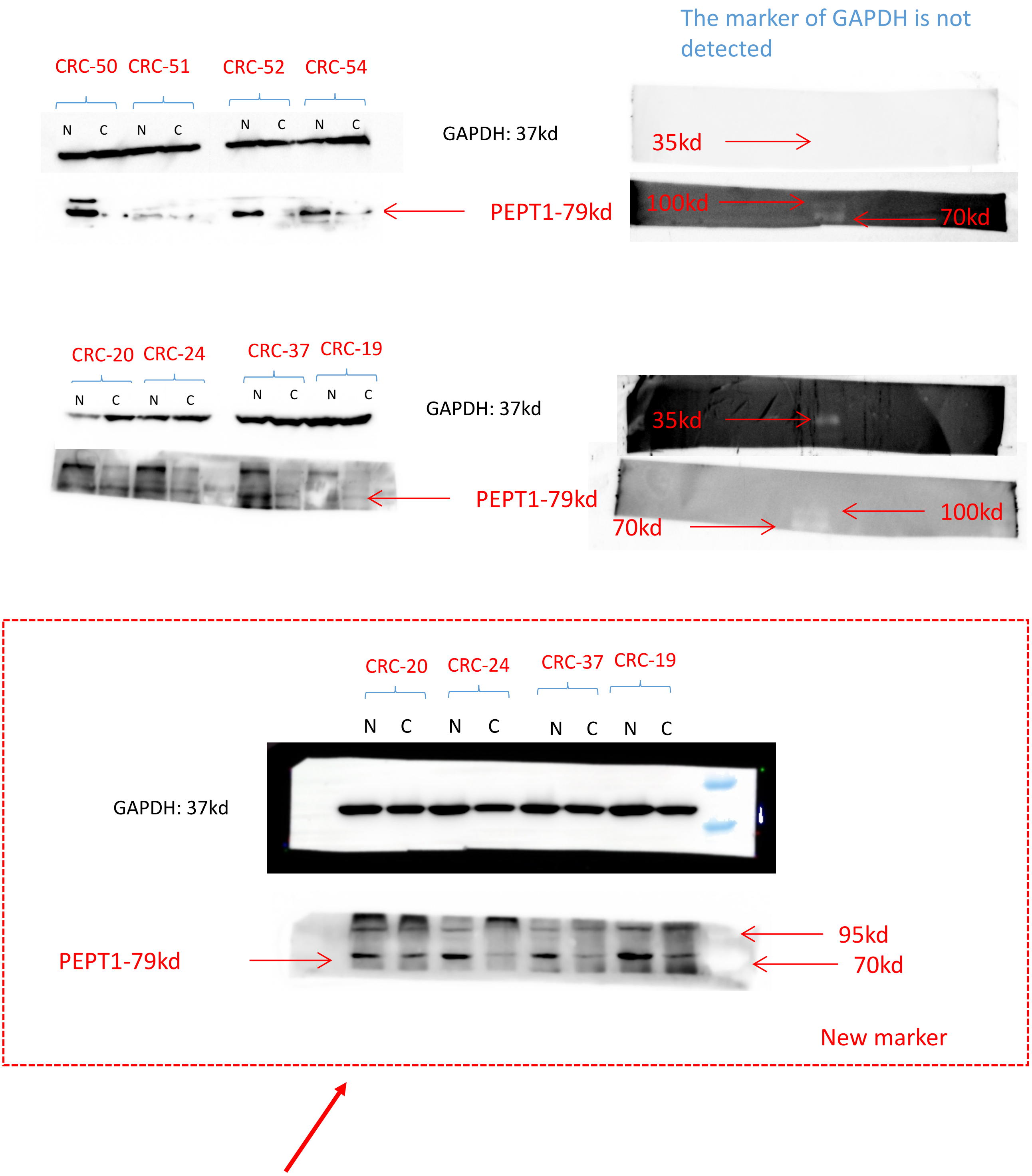

We used different instrument to exposure the western blot bands of these 4 paired CRC tissues in contrast to previous CRC tissues, which is the same as all the bands of CRC cells.

Figure2B

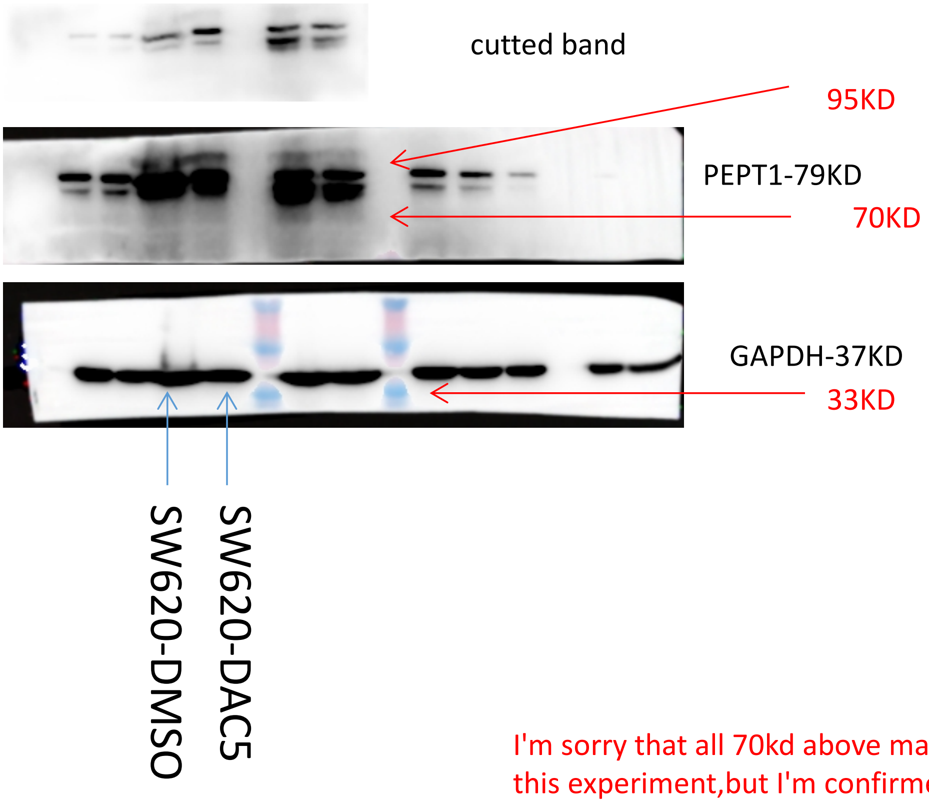

I'm sorry that all 70kd above marker degraded in this experiment,but I'm confirmed the location.

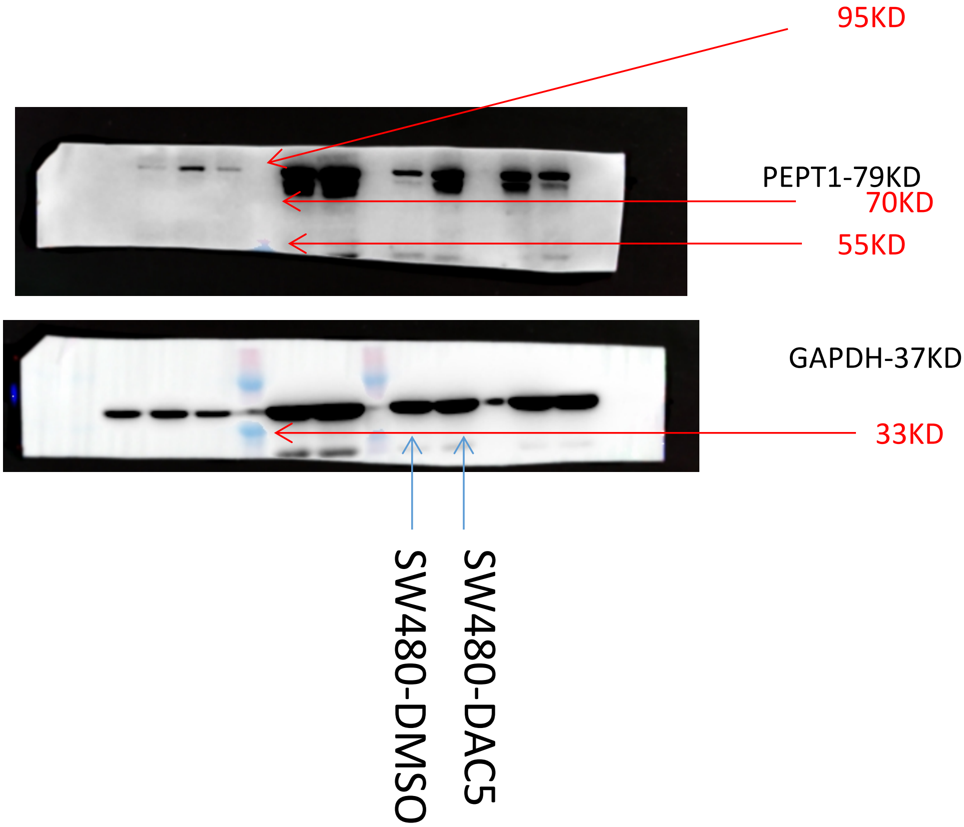

Figure2E

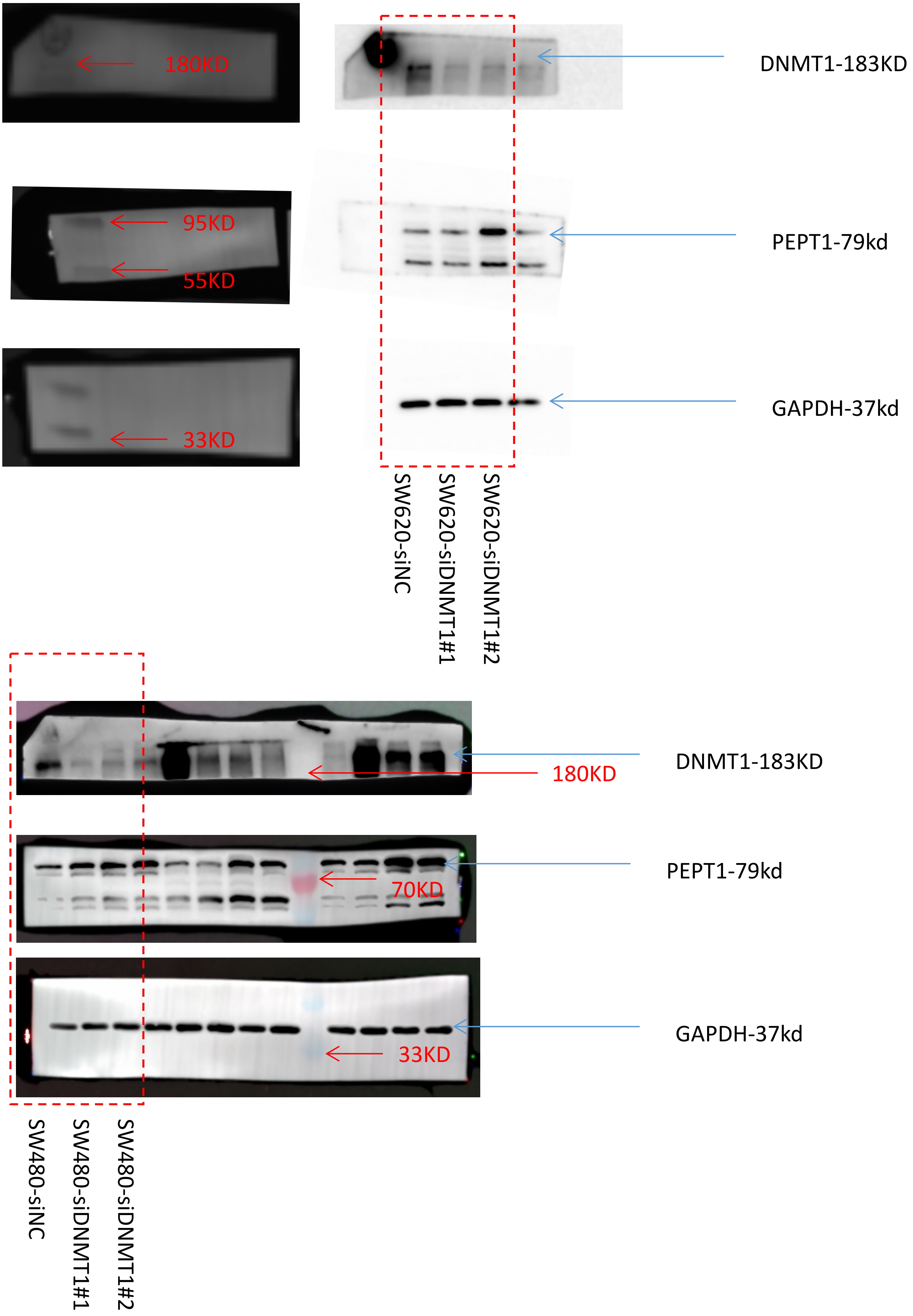

180KD
DNMT1-183KD
95KD
55KD
PEPT1-79kd
33KD
GAPDH-37kd
SW620-siNC
SW620-siDNMT1#1
SW620-siDNMT1#2
DNMT1-183KD
180KD
PEPT1-79kd
70KD
GAPDH-37kd
33KD
SW480-siNC
SW480-siDNMT1#1
SW480-siDNMT1#2

Figure4B

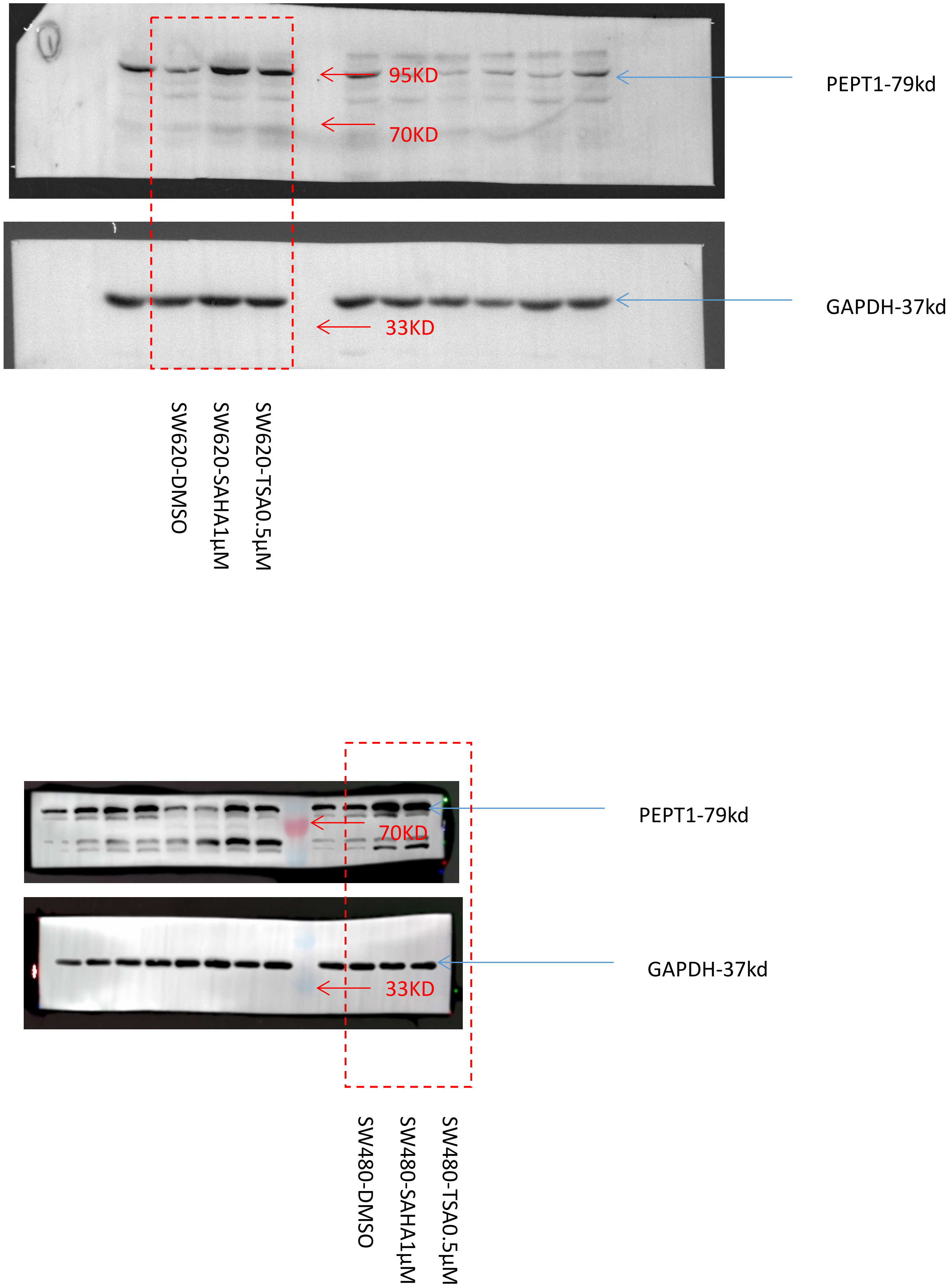
95KD
70KD
PEPT1-79kd
33KD
GAPDH-37kd
SW620-DMSO
SW620-SAHA1μM
SW620-TSA0.5μM
PEPT1-79kd
70KD
GAPDH-37kd
33KD
SW480-DMSO
SW480-SAHA1μM
SW480-TSA0.5μM

Figure4D

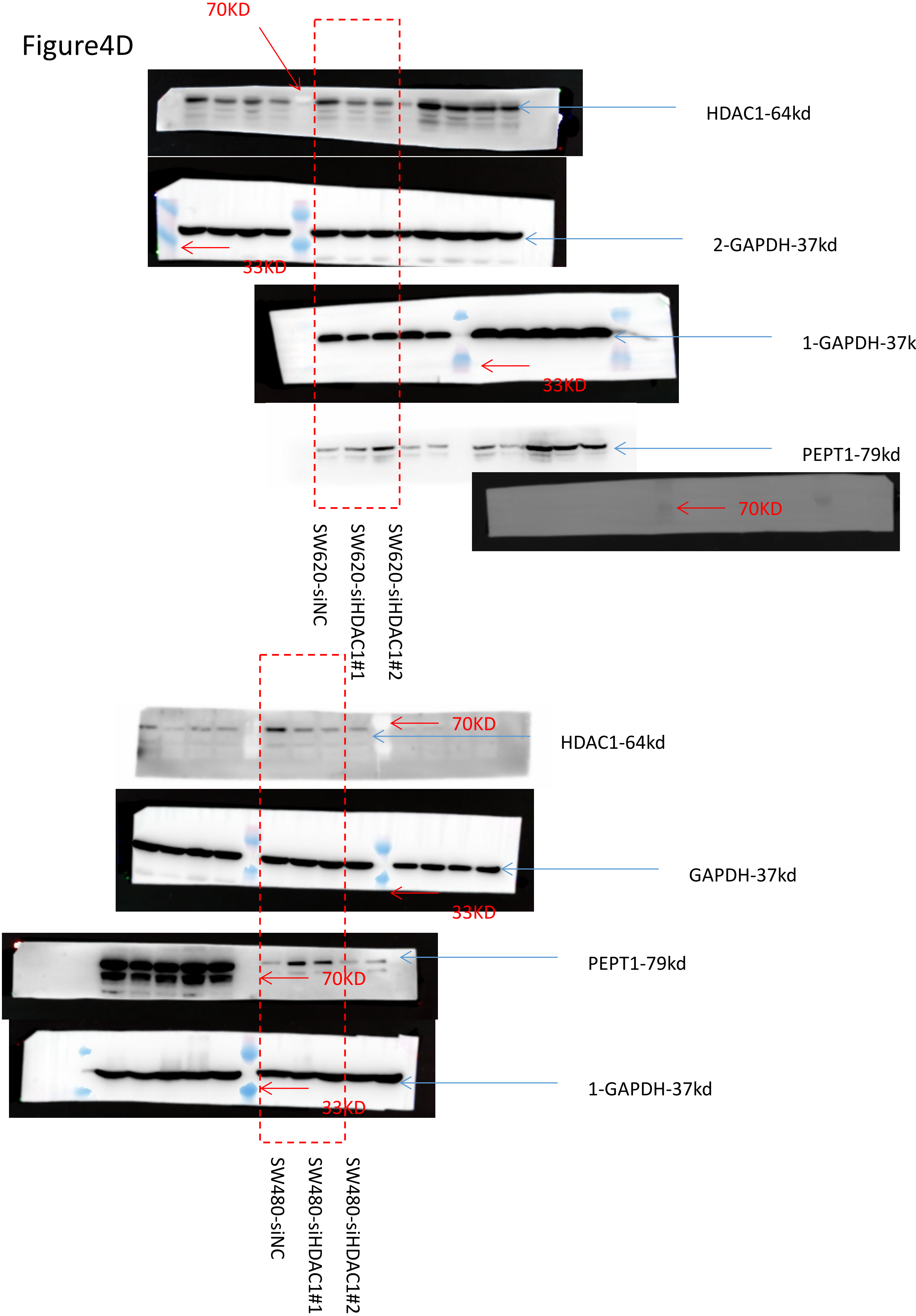
70KD
HDAC1-64kd
2-GAPDH-37kd
33KD
1-GAPDH-37k
33KD
PEPT1-79kd
70KD
SW620-siNC
SW620-siHDAC1#1
SW620-siHDAC1#2
70KD
HDAC1-64kd
GAPDH-37kd
33KD
PEPT1-79kd
70KD
1-GAPDH-37kd
33KD
SW480-siNC
SW480-siHDAC1#1
SW480-siHDAC1#2

Figure5D

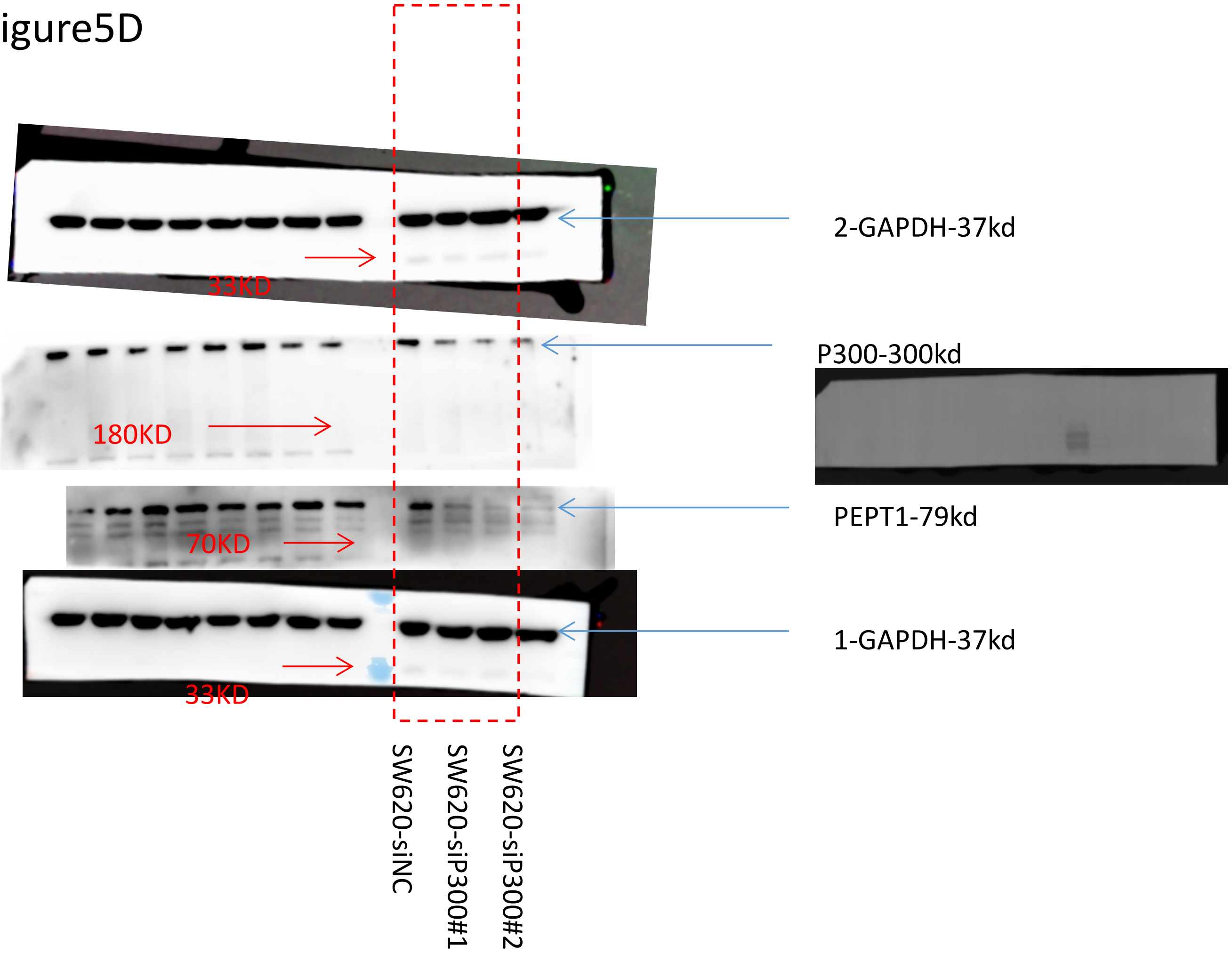

2-GAPDH-37kd
33KD
P300-300kd
180KD
PEPT1-79kd
70KD
1-GAPDH-37kd
33KD
SW620-siNC
SW620-siP300#1
SW620-siP300#2

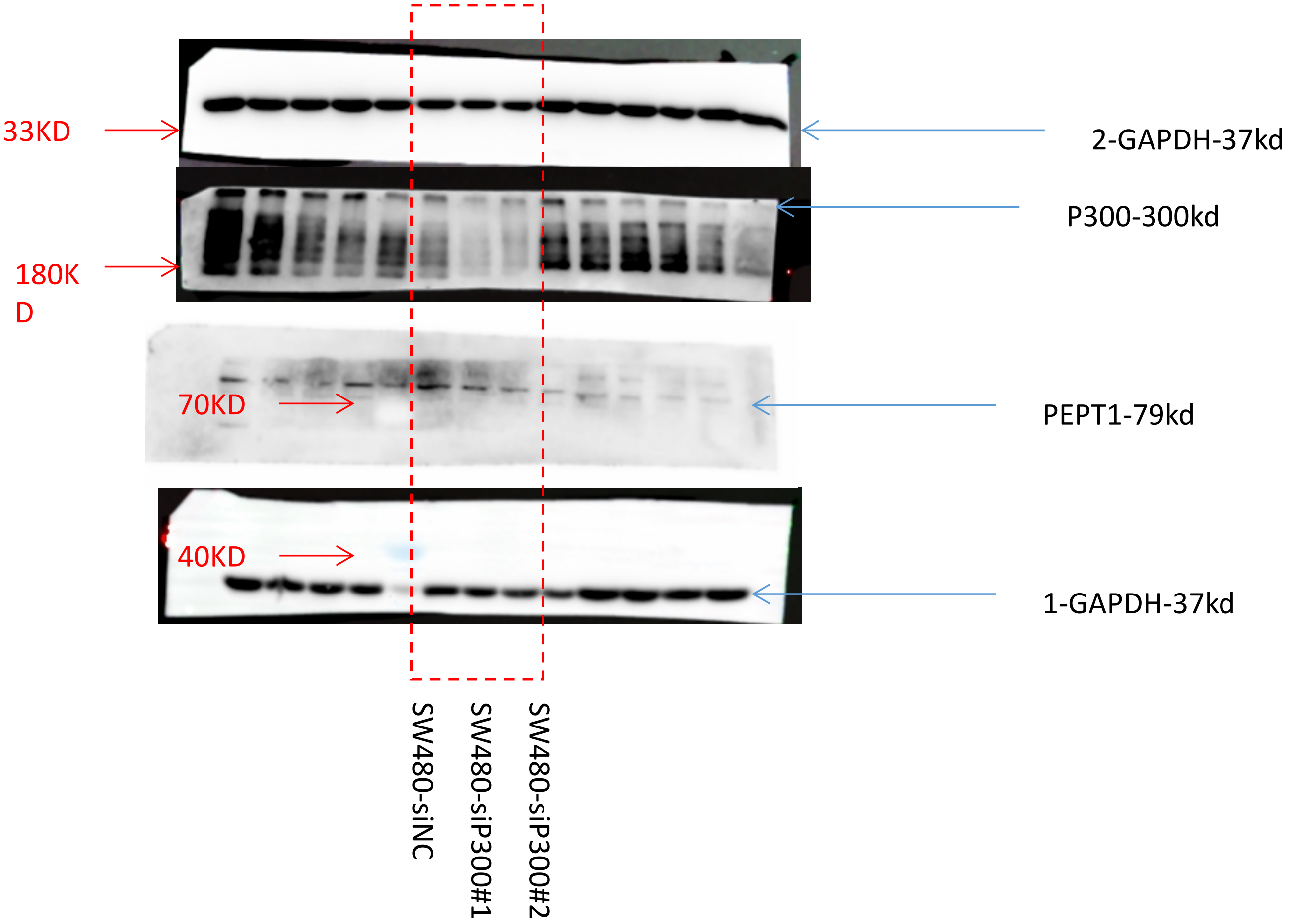

33KD
2-GAPDH-37kd
180K
D
P300-300kd
70KD
PEPT1-79kd
40KD
1-GAPDH-37kd
SW480-siNC
SW480-siP300#1
SW480-siP300#2

FigureS6B

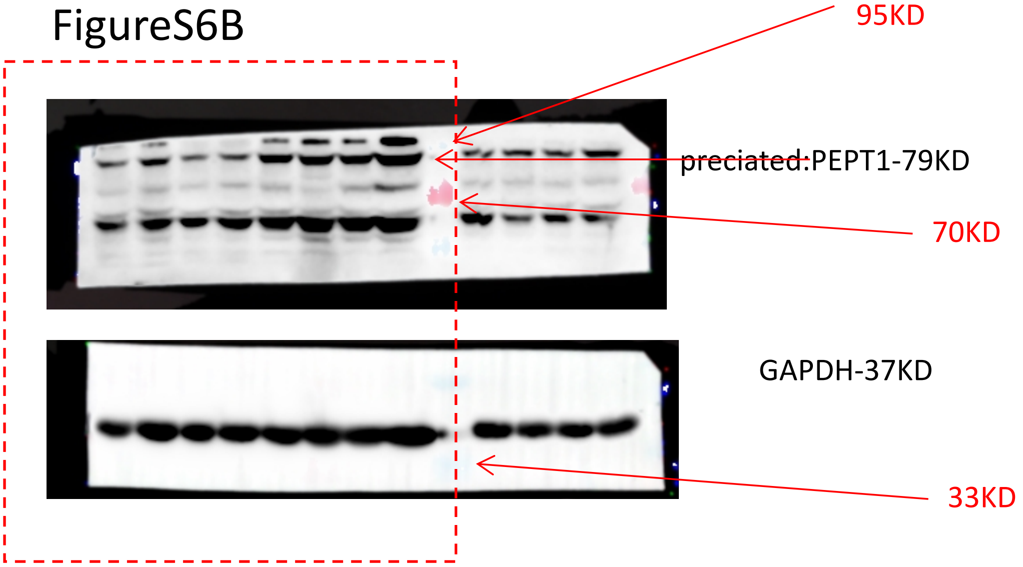

Sw620-xenografts: NC-3 NC-4 Ube-4 Ube-5 DAC-1 DAC-3 D+U-2 D5+U Marker

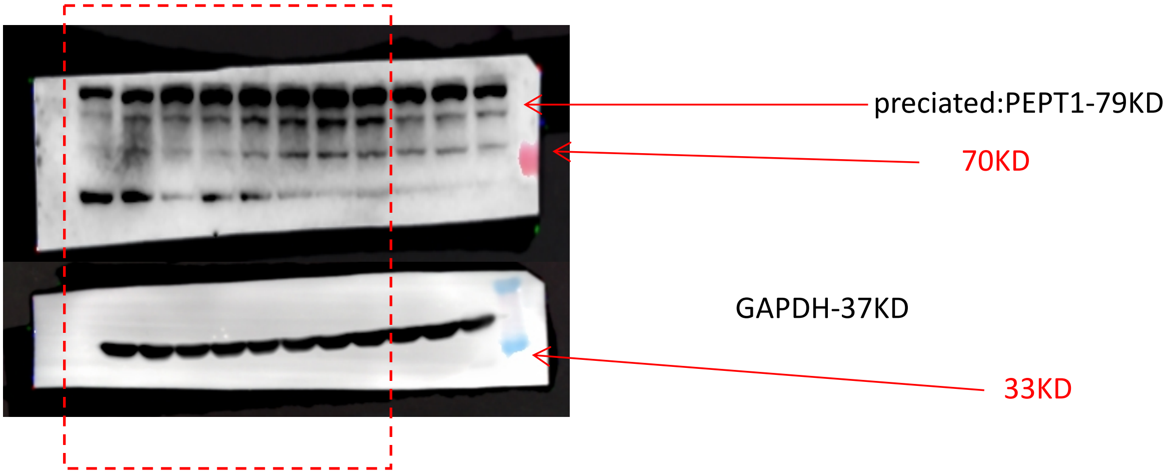

SW480-xenografts：NC-2 NC-5 Ube-1 Ube-3 DAC-1 DAC-3 D+U-2 D+U-4
